# Structure of the type IV secretion system in different strains of *Anaplasma phagocytophilum*

**DOI:** 10.1186/1471-2164-13-678

**Published:** 2012-11-29

**Authors:** Basima Al-Khedery, Anna M Lundgren, Snorre Stuen, Erik G Granquist, Ulrike G Munderloh, Curtis M Nelson, A Rick Alleman, Suman M Mahan, Anthony F Barbet

**Affiliations:** 1 Department of Infectious Diseases and Pathology, College of Veterinary Medicine, University of Florida, Gainesville, FL, USA; 2 Department of Production Animal Sciences, Section of Small Ruminant Research, Norwegian School of Veterinary Science, Sandnes, Norway; 3 Department of Entomology, University of Minnesota, St Paul, MN, USA; 4 Physiological Sciences, College of Veterinary Medicine, University of Florida, Gainesville, FL, USA; 5 Pfizer Animal Health, Kalamazoo, MI, USA

**Keywords:** *Anaplasma*, *phagocytophilum*, *Rickettsiales*, T4SS, Comparative genomics

## Abstract

**Background:**

*Anaplasma phagocytophilum* is an intracellular organism in the Order *Rickettsiales* that infects diverse animal species and is causing an emerging disease in humans, dogs and horses. Different strains have very different cell tropisms and virulence. For example, in the U.S., strains have been described that infect ruminants but not dogs or rodents. An intriguing question is how the strains of *A*. *phagocytophilum* differ and what different genome loci are involved in cell tropisms and/or virulence. Type IV secretion systems (T4SS) are responsible for translocation of substrates across the cell membrane by mechanisms that require contact with the recipient cell. They are especially important in organisms such as the *Rickettsiales* which require T4SS to aid colonization and survival within both mammalian and tick vector cells. We determined the structure of the T4SS in 7 strains from the U.S. and Europe and revised the sequence of the repetitive *virB6* locus of the human HZ strain.

**Results:**

Although in all strains the T4SS conforms to the previously described split loci for *vir* genes, there is great diversity within these loci among strains. This is particularly evident in the *virB2* and *virB6* which are postulated to encode the secretion channel and proteins exposed on the bacterial surface. *VirB6*-*4* has an unusual highly repetitive structure and can have a molecular weight greater than 500,000. For many of the *virs*, phylogenetic trees position *A*. *phagocytophilum* strains infecting ruminants in the U.S. and Europe distant from strains infecting humans and dogs in the U.S.

**Conclusions:**

Our study reveals evidence of gene duplication and considerable diversity of T4SS components in strains infecting different animals. The diversity in *virB2* is in both the total number of copies, which varied from 8 to 15 in the herein characterized strains, and in the sequence of each copy. The diversity in *virB6* is in the sequence of each of the 4 copies in the single locus and the presence of varying numbers of repetitive units in *virB6*-*3* and *virB6*-*4*. These data suggest that the T4SS should be investigated further for a potential role in strain virulence of *A*. *phagocytophilum*.

## Background

*Anaplasma phagocytophilum* is a tick-borne pathogen in the Order *Rickettsiales* that is increasingly recognized as a cause of disease in humans and animals world-wide [1,2]. It causes the potentially fatal disease of human granulocytic anaplasmosis, which typically manifests as a flu-like illness accompanied by leukopenia, thrombocytopenia and anemia. It was initially recognized in the early 1990's when patients from Wisconsin and Minnesota developed febrile illness following a tick bite [3]. Since that time the number of human cases has increased annually; between 2000 and 2007 the reported incidence in the U.S. increased from 1.4 to 3.0 cases/million persons/year [4]. The case fatality rate was 0.6% and the hospitalization rate was 36%. In Massachusetts during the 2009 transmission season there were 33 confirmed cases with 14 (42%) requiring hospitalization [5]. The human disease is also present in Europe and Asia [2]. A recent study of 83 *A*. *phagocytophilum*-infected patients in China reported a mortality rate in this cohort of 26.5% [6]. In the U.S., there has been a parallel increase in cases of the disease [7] and seroprevalence [8] in dogs in the eastern and upper Midwestern states. The tick vectors in the U.S. are *Ixodes scapularis* and *Ixodes pacificus* and wild rodents are the main reservoirs of human infections. *A*. *phagocytophilum* also infects numerous other mammalian species including ruminants, horses, cats, and bears and the symptoms are extremely variable, with some mammalian species exhibiting acute disease and others only persistent asymptomatic infections [9,10]. For example, *A*. *phagocytophilum* strains isolated from deer in the U.S. can have a slightly different 16S rRNA sequence and be uninfective to mice and it is thought, humans [11-13]. In Europe, this agent has been known to cause disease of ruminants for >100 years, yet there have been few human infections [14]. The genome sequence is available for a single strain of *A*. *phagocytophilum* derived from an infected human in the U.S. and it is apparent that, although this strain lacks Type II, III, V and VI secretion systems, a Type IV secretion system (T4SS) is present [15]. As in other members of the *Rickettsiales*, the T4SS of *A*. *phagocytophilum* is organized differently from most gram-negative bacteria with the component *vir* genes distributed between three major genome locations [16].

The T4SS typically encodes a membrane-spanning multiprotein complex that forms a transmembrane channel through which solutes can pass into host cells. It can mediate transfer of DNA and proteins into eukaryotic host cells, interfere with host signaling, and is essential for the survival of intracellular bacteria [17]. In *A*. *phagocytophilum*, which preferentially colonize neutrophilic white blood cells, it is thought that the T4SS secretes virulence factors that are responsible for subverting innate immunity and inhibiting host cell apoptosis [16]. Interestingly, there appears to be differential transcription of the T4SS in ticks and in the mammalian host with *virB6* and *virB9* upregulated during infection of human neutrophils and different *virB2* paralogs expressed in mammalian and tick cells [18]. There is evidence that VirB2, VirB6 and VirB9 are exposed on the outer membrane surface in the *Rickettsiales *[18-20], which has stimulated interest in their potential use as vaccine candidates. This possibility has been investigated more extensively in the related organism *Anaplasma marginale *[21-25]. In *A*. *marginale*, unlike many other surface-exposed proteins, the T4SS proteins are conserved between strains [26]. Also, cattle immunized with outer membranes and protected against challenge infection respond with IgG and T cells to Vir proteins, notably VirB2, VirB9 and VirB10. To date, only two T4SS substrates have been identified and partially characterized in *A*. *phagocytophilum*: the ankyrin repeat domain-containing protein, AnkA, and the *Anaplasma* translocated substrate 1, Ats-1. AnkA translocates to the host nucleus and interacts with DNA [27,28], while Ats-1 is imported into the mitochondria where it is proposed to interfere with the induction of apoptosis [29].

In this study, we compared the structure and diversity of the T4SS in different strains of *A*. *phagocytophilum* infecting humans, dogs, rodents and ruminants. Most diversity was found in the proteins thought to be surface-exposed, which may be associated with the different virulence and cell invasion properties of this species.

## Results and discussion

**Figure 1:**
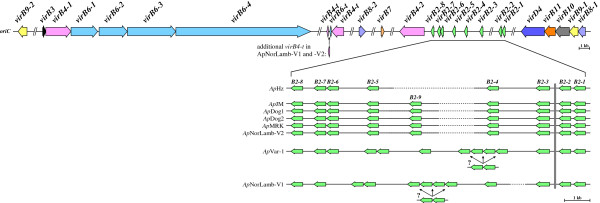
**Distribution and content of *vir *gene clusters in eight diverse *A. phagocytophilum *strains.** Top panel. Schematic representation of all *vir* loci (colored arrows) showing the three conserved gene cluster islands (see text). *VirB*-*7*, *virB8*-*2* and *virB9*-*2* are not part of *vir* gene clusters, but their location relative to surrounding genes is also highly conserved among strains. A small cluster comprising truncated (t) *virB6* and *virB4* gene fragments is present in all strains, but the Norwegian lamb strains have one additional *virB4*-*t*. Bottom panel. Magnification of the *virB2* gene cluster. Numbering of paralogs 1–8 is based on the original *Ap*HZ annotated genome (GenBank CP000235). Artificial gaps (stippled lines) were introduced to allow alignment of the more spatially conserved paralogs *B2*-*1*, *2*–*2* and *2*–*3* at one end, and *B2*-*7* and *2*–*8* at the other end of the cluster. With the exception of *virB2*-*9*, lacking in *Ap*HZ, the number and arrangement (but not necessarily sequence) of *virB2* genes is highly conserved in all but the US ruminant *Ap*Var-1 and *Ap*NorLamb-V1, which have several additional *virB2* genes. In both strains a sub-cluster of 6 distinct genes was present. Due to the repetitive nature of sequences in this region, combined with the relatively short length of 454 reads (≤550 bp), their placement could not be confidently ascertained (highlighted by arrows and ‘?’). Maps are drawn to scale. Double lines designate interruption in sequences. Genes belonging to the same grouping have the same color. *oriC*; origin of replication.

The *vir* loci were sequenced in eight strains of *A*. *phagocytophilum*; seven of these were strains for which previous structural information was not available and included organisms originally isolated from U.S. dogs (*Ap*Dog1, *Ap*Dog2), a rodent (*Ap*JM), a horse (*Ap*MRK), the ruminant *Ap* variant 1 strain (*Ap*Var1) and two strains from Norwegian sheep (*Ap*NorV1, *Ap*NorV2). The human HZ strain was also resequenced, as optical mapping had suggested a possible error in the previously sequenced *virB64* locus. The data indicated considerable diversity in the individual *vir* loci between strains that will be discussed below. In all strains, however, as noted previously [20,30], the *vir* loci were distributed mainly in three gene clusters comprising: *virB81*, *virB91*, *virB10*, *virB11* and *virD4*; *virB2*′s and *virB42*; and *virB3*, *virB41*, and the four *virB6* paralogs (Figure 1). These three loci may each be transcribed polycistronically [31], although it is clear that T4SS structure in the *Rickettsiales* is unique and more complex than initially thought. The number of *virB2* paralogs was different between strains with the human HZ strain having the least (8 total paralogs) and the ruminant strains having the most (up to15 total paralogs). The description of the T4SS components presented here follows the functional classification described by Alvarez-Martinez and Christie [20].

### Inner membrane channel/scaffold subunits: VirB3, VirB6, VirB8 and VirB10

The most conserved of these subunits are VirB3, VirB8 and VirB10, with few differences between strains. VirB3 has been linked in *Agrobacterium tumefaciens* with pilus assembly and substrate translocation [32,33]. It is absolutely conserved between strains with no amino acid changes and conforms to the typical VirB3 structure. Two alpha-helical domains for insertion into the cytoplasmic membrane are strongly predicted by TMpred. VirB8, proposed to function as a nucleation factor during the assembly of T4SS [34,35], is also well conserved, particularly VirB8-1 in the polycistronic transcription locus (one amino acid change between all strains). VirB10, proposed to function as a scaffold across the entire cell envelope [36], is also generally well-conserved with the exception of one ruminant strain, *Ap*NorLamb-V1, which has 31 amino acid substitutions with respect to *Ap*HZ (data not shown). However, all *A*. *phagocytophilum* VirB10^′^s, including *Ap*NorLamb-V1, have two strongly predicted transmembrane domains, which supports their function as membrane scaffolding subunits in these organisms.

**Figure 2:**
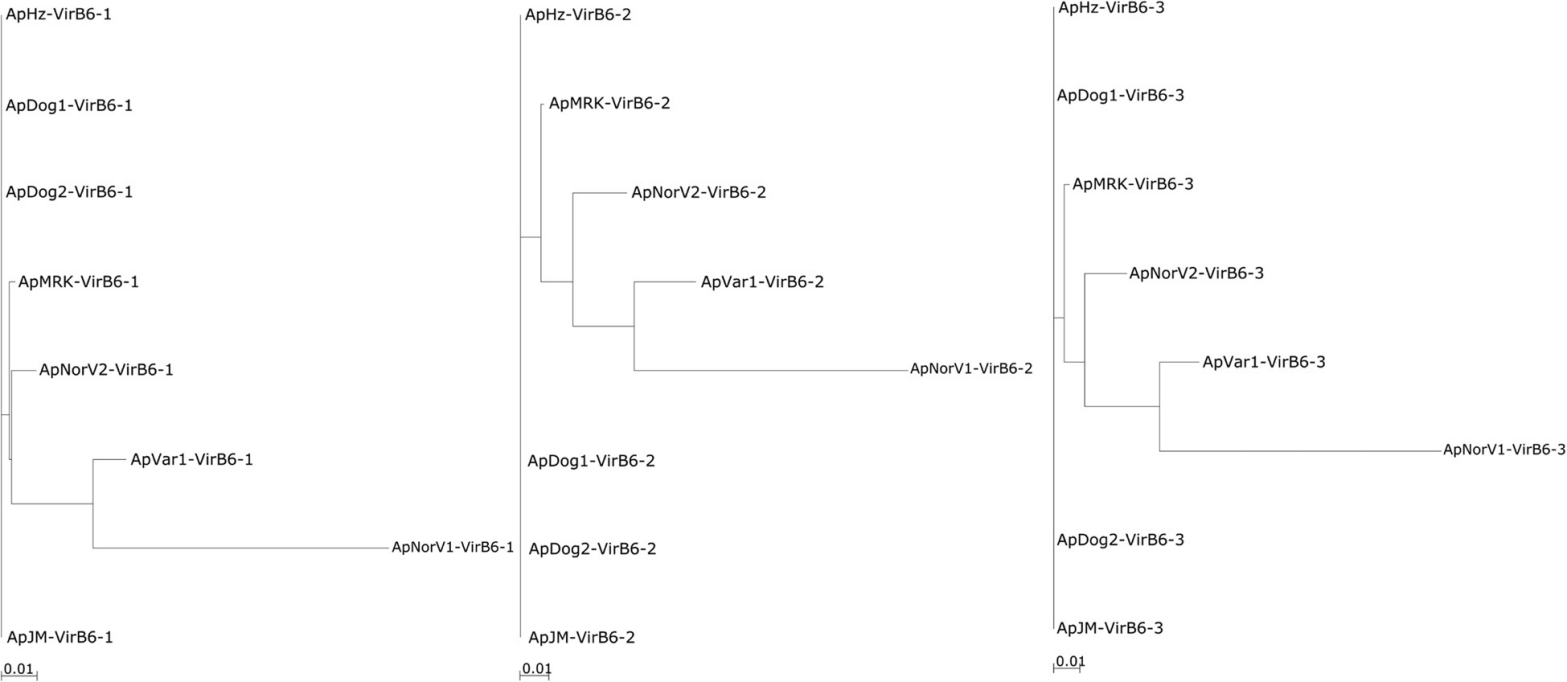
**Phylogenetic tree to show the relationship of syntenic VirB6 proteins from different strains of *A*. *phagocytophilum*.** A scale bar is shown underneath representing the number of amino acid substitutions/site.

**Figure 3:**
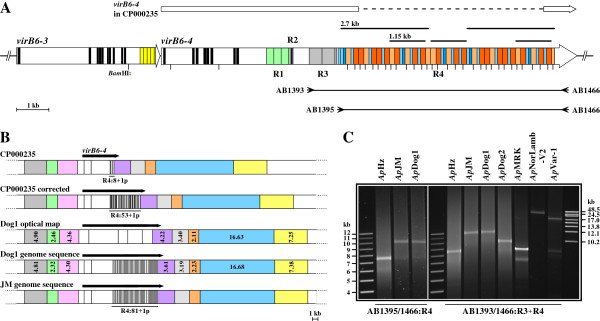
**The 3**^′ ^**end of *A. phagocytophilum virB6*-*4 *genes is composed of an unusually large tandem repeat region, which exhibits dramatic variability among strains.A**. Map of the human HZ strain *virB6*-*3* and *virB6*-*4* genes, highlighting the location and structure of several repeat regions (R1-R4). The most variability occurred in R4; this region is 5.88 kb larger than previously reported for the *Ap*HZ genome (CP000235). The original sequence is diagrammed above the map, with the dashed line representing the segment missing in CP000235. Larger repeated R4 segments of 2.7 kb and 1.15 kb are indicated above. Vertical black bars within each gene designate segments encoding predicted transmembrane domains. BamHI sites, of which there is one in all R4 type 2 repeats (see Figure S2B), are indicated. Also shown are the positions of PCR primers used in C. **B**. BamHI genomic maps depicting the *virB6*-*4* locus (black arrows).The segment encompassing R4 is highlighted below each respective map. In the regions outside the *virB6*-*4* locus, corresponding BamHI fragments are shown in the same color. Overall, the optical map sizes were in good agreement with the actual sizes, except within R4. This is attributed to the limitation of optical mapping in resolving fragments <2 kb. Despite these discrepancies, the cumulative size of the genomic region encompassing *virB6*-*4* in the optical map is in close agreement with that in the *Ap*Dog1 genome sequence. **C**. The variability in size of PCR products spanning *virB6*-*4* repeat regions R4 and R3/R4 in diverse *A*. *phagocytophilum* strains.

Of these inner membrane channel subunits, the data on VirB6 are the most interesting. All VirB6 subunits that have been described possess a highly hydrophobic membrane domain including five or more predicted transmembrane domains [20]. Some VirB6 proteins also have an extended C-terminal hydrophilic domain that has been proposed to protrude through the T4SS into the target cell, or may be proteolytically released from the N-terminal domain and then translocated into the target cell. Evidence has been obtained for surface exposure of extended VirB6 in some *Rickettsiales *[37]. Of all the membrane channel subunits, the most sequence diversity between *A*. *phagocytophilum* strains was in the four VirB6 paralogs (Figure 1). Although there were no amino acid changes in the VirB6-1, VirB6-2 and VirB6-3 paralogs between human, dog and rodent strains, the ruminant and horse strains had numerous substitutions throughout each molecule, agreeing with the closer evolutionary relationship between strains infecting humans and dogs in the U.S. (Figure 2). Furthermore, major differences in repeat number and sequence were found in the C-terminal repeat region of VirB6-3 (yellow boxes in Figure 3A and Additional file 1: Figure S1) in ruminant and horse strains, with the horse strain showing the least variability from *Ap*HZ.

The only amino acid differences detected between the human, dog and rodent strains were in the VirB6-4 subunit. *VirB6*-*4* in these strains contains four repeat regions (R1-R4 in Figure 3A) and variability in repeat number, order and sequence were found mainly in R3 and R4 (Additional file 2: Figure S2). Within R1 (Figure 3A), the only difference detected was in *Ap*Dog2 which had 4 and 1 partial of 231 bp repeat units (data not shown), compared to 3 and 1 partial repeats in the *Ap*Dog1, *Ap*JM and *Ap*HZ *virB6*-*4* R1. Optical mapping of the Dog1 genome and comparison with *Ap*HZ suggested that the sequence obtained previously for the human HZ strain *virB6*-*4* was incorrect (Figure 3B). This was confirmed by PCR and sequencing, and mapped specifically to the 3^′^-most R4 region (Figure 3C). Because of its size and unusual composition it was only possible to resolve this sequence using the long read-length Pacific Biosciences technology (see Methods). The corrected *virB6*-*4* R4 of *Ap*HZ, totaling 6.89 kb, differed from the original by 5.88 kb of additional sequence composed exclusively of 84 bp [type 1, a and b (T1a, T1b); light/dark blue boxes, respectively, in Figure 3A]and 162 bp [type 2, a and b (T2a, T2b); light/dark orange boxes, respectively, in Figure 3A] repeat units, giving a complex repeat structure containing 53 and 1 partial repeat units compared to 8 and 1 partial in the original sequence. Further, the 5^′^- and 3^′^-most 2.7 kb of this complex structure are identical in sequence, and the 3^′^-most 1.15 kb of each of these segments is repeated again in the center of R4 (Figure 3A and Additional file 2: Figure S2). Although the possibility exists that the *Ap*HZ population from which we isolated gDNA differs within the *virB6*-*4* R3/R4 repeat regions from the population used to generate CP000235, the fact that all strains investigated herein presented expansive R3/R4 regions (Figure 3C) would contradict that. Instead, it is more plausible that the existence of 2.7 kb of identical repeats at the ends of the *Ap*HZ R4 may have lead to the excision of most of its sequence during construction/propagation of those libraries. Interestingly, *virB6*-*4* R3 and R4 were identical both in size and sequence in the Dog1 and rodent strains despite differing markedly from the HZ and Dog2 strain regions (Additional file 2: Figure S2A). Within R3, these strains had 2 additional 405 bp repeats compared to *Ap*HZ and one more compared to the Dog2 strain. However, differences between strains were most dramatic within R4. Not only was this region in *Ap*Dog1/*Ap*JM 2.87 kb larger than in *Ap*HZ bringing the total number of repeats to 81 and 1 partial, but intriguingly, the repeat pattern was completely unrelated to that in the HZ strain. Also, the Dog1 and rodent strain R4 lacked T1b repeat units, while having a third type 2 repeat variant, namely T2c, which differed from T2b by 1 SNP and a 12 bp deletion (Additional file 2: Figure S2). Partial analysis of the *Ap*Dog2 454 reads spanning R4 (estimated at ~8 kb by PCR; Figure 3C) showed that the order of the 5^′^- and 3^′^-most three repeat units differed from either the HZ or Dog1/rodent strain R4 repeat patterns (Additional file 2: Figure S2A). Notably, our preliminary analyses of the horse and ruminant 454 reads suggest the absence of distinct R3 and R4 regions in *virB6*-*4* in these strains. Rather, the few repeat units identified to date appear to be a combination of R3 and R4 repeats (data not shown). It is also unclear if the ~17 kb and ~25 kb PCR products generated with primers AB1393/1466 in *Ap*Var-1 and *Ap*NorLamb-V2, respectively (Figure 3C), are composed mainly of repeats, or alternatively if a fifth *virB*-*6* gene paralog exists in these strains. Taken together, the data presented here clearly demonstrate the extreme variability of the T4SS VirB6-4 subunit among *A*. *phagocytophilum* strains. Although the differences between the more closely related human, dog and rodent US strains were mainly within repeat-laden regions, the fact that an extensive, distinct repeat pattern was maintained in two strains would speak against the possibility that the variability may be attributed solely to the highly recombinogenic nature of such structures. Worth noting, Camp Ripley, where the infected jumping mouse was captured (2001) is only ~20 miles away from the city of Baxter, MN, where Dog1 resides. Although there are no records of where this dog may have actually acquired the infection, it presented with severe clinical disease in 2007.

**Figure 4:**
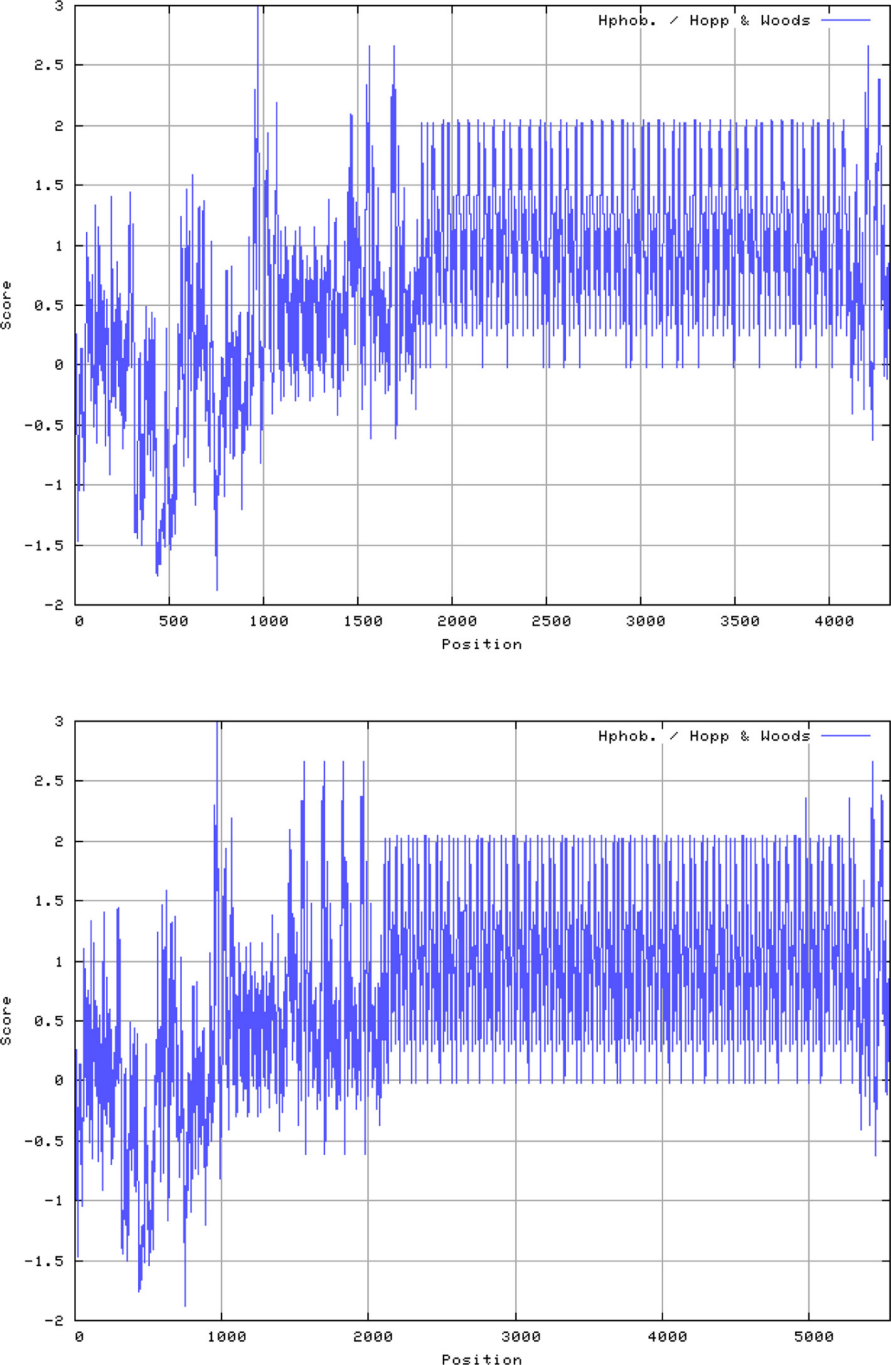
**Hydrophobicity plots of VirB6-4 proteins from *A. phagocytophilum *HZ (top) or Dog 1 (bottom) strains.**

The unusual structure and likely antigenicity of the C-terminal region of the *A*. *phagocytophilum* VirB6-4^′^s is apparent in hydrophobicity plots (Figure 4). What specific properties these distinct repeat patterns may confer onto each strain awaits functional analysis of these proteins in *A*. *phagocytophilum*. The corrected VirB6-4 translated protein had a predicted molecular weight of 470,695 Da containing 4,322 amino acid residues compared to molecular weights of 90,742, 103,204 and 158,321 Da for the HZ strain VirB6-1, VirB6-2 and VirB6-3, respectively. Interestingly, the predicted acidity of the VirB6^′^s also increased from VirB6-1 to VirB6-4 (pI’s of 8.4, 6.8, 5.1 and 4.0 for the *Ap*HZ VirB6-1, VirB6-2, VirB6-3 and VirB6-4, respectively). The *Ap*Dog1/*Ap*JM VirB6-4 polypeptides had a predicted molecular weight of 603,529 Da containing 5,550 amino acids, and a pI of 3.96. Despite these dissimilarities, at least eight transmembrane segments were predicted for all VirB6 paralogs.

### Periplasmic/outer membrane channel subunits: VirB2, VirB7 and VirB9

Several other T4SS subunits contribute to the secretion channel across the periplasm and outer membrane. VirB7 subunits are typically small lipoproteins that may stabilize VirB9 [38,39]. In *A*. *phagocytophilum* strains a putative VirB7 is absolutely conserved between strains and may be lipid modified through an N-terminal cysteine on the mature molecule. VirB9 is hydrophilic and also localizes to the periplasm and outer membrane. In *A*. *tumefaciens* the C-terminal region of VirB9 is part of the outer membrane protein channel and is surface accessible [40]. There is also evidence for surface exposure of VirB9 in *Ehrlichia chaffeensis* and *A*. *phagocytophilum*[18,19,41]. VirB9-1, which is encoded on the polycistronic *virB81virD4* transcript [31], has a strongly predicted signal peptide and two transmembrane helices. Of all the potentially exposed components of the T4SS, VirB9 of *A*. *phagocytophilum* appears to be the least diverse among strains. There are some amino acid substitutions in ruminant and horse strains (2–6 total compared to *Ap*HZ) but in the other strains VirB9^′^s are unchanged (data not shown).

**Figure 5:**
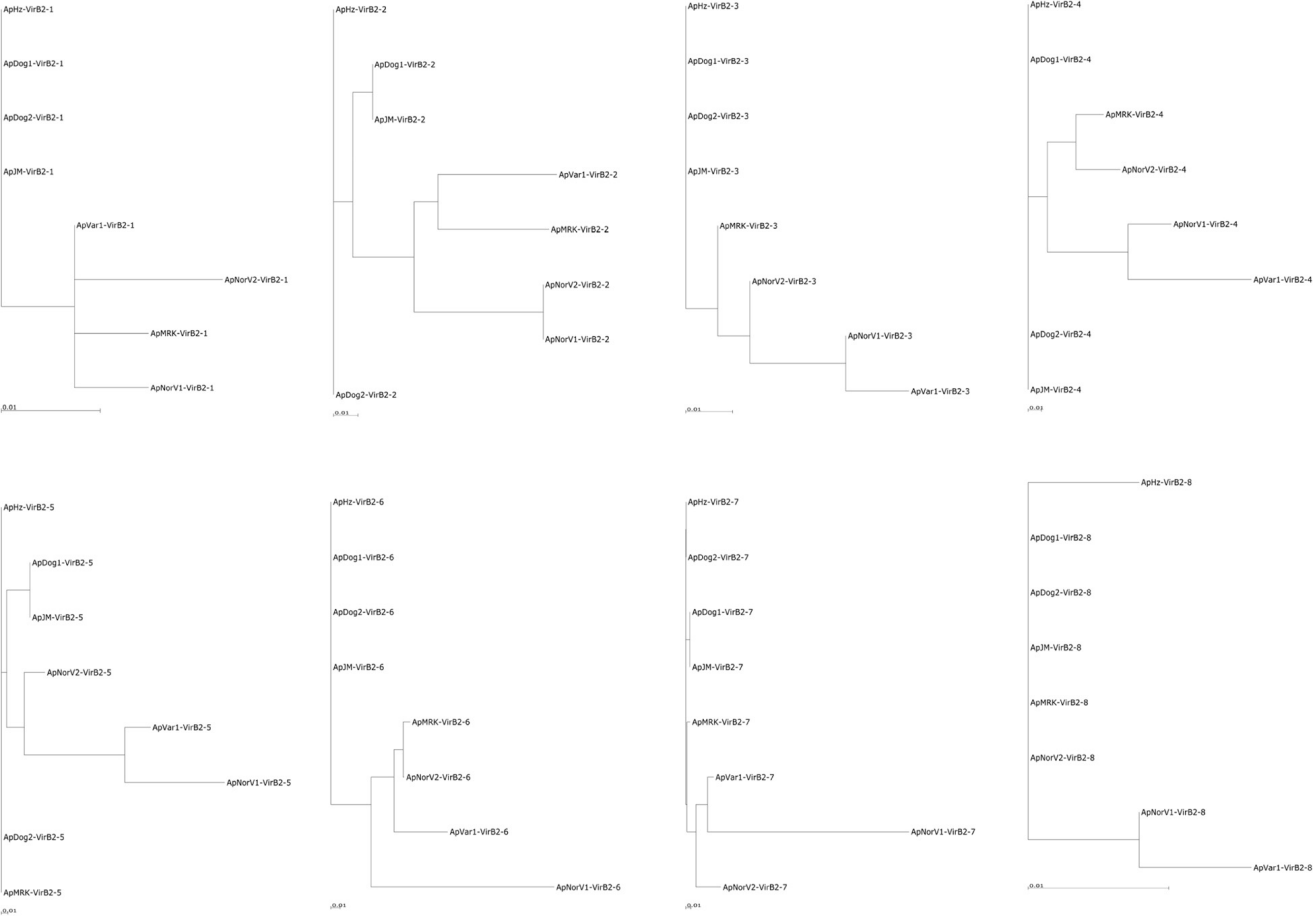
**Phylogenetic trees to show the relationship of syntenic VirB2 proteins from different strains of *A. phagocytophilum.***

**Figure 6:**
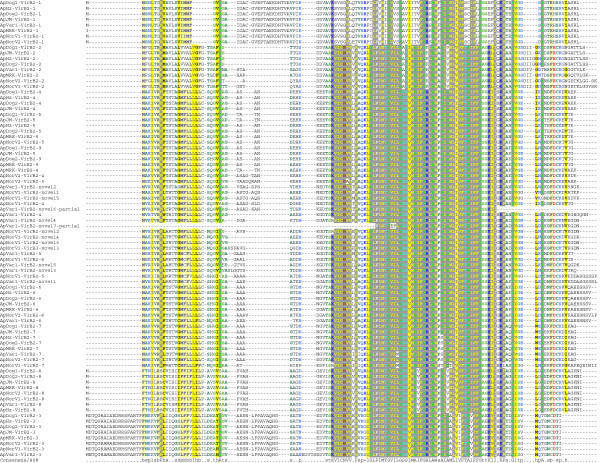
**Multiple sequence alignment of VirB2 amino acid sequences from different strains of *A. phagocytophilum*.**

Unlike VirB9^′^s, VirB2^′^s are the most diverse of all T4SS subunits in *A*. *phagocytophilum*, in terms of both copy number and sequence. VirB2 proteins are typically constituents of pili and of the secretion channel and their diversity in *Anaplasma* suggests the possibility of exposed, antigenically variable structures. In *A*. *marginale*, VirB2 is expressed together with the major outer membrane protein MSP3 on a sequence-variable polycistronic transcript [25,42]. The mechanism of expression in *A*. *phagocytophilum* is not known. VirB2^′^s of other genera are typically small hydrophobic proteins with a long signal peptide sequence and two hydrophobic alpha helices for integration into the cytoplasmic membrane. This also appears to be the case for *A*. *phagocytophilum*. The VirB2 paralogs in the different strains are predicted to have two hydrophobic alpha-helices of lengths 22+/−3 and 20+/−0.2 amino acids and signal peptides of length 27+/−2 amino acids. This is true despite their sequence diversity (Figure 5). As with many other T4SS components, the ruminant and horse strains are more distant taxonomically in VirB2 sequence compared to VirB2^′^s of human and dog strains. Alignment of all VirB2 paralogs and orthologs shows that sequence diversity is primarily localized to two hypervariable regions either preceding an N-terminal cysteine or close to the C-terminus (Figure 6). This is similar to the hypervariable regions found among VirB2 paralogs of *A*. *marginale*[25].

### Energetic subunits: VirB4 and VirB11

**Figure 7:**
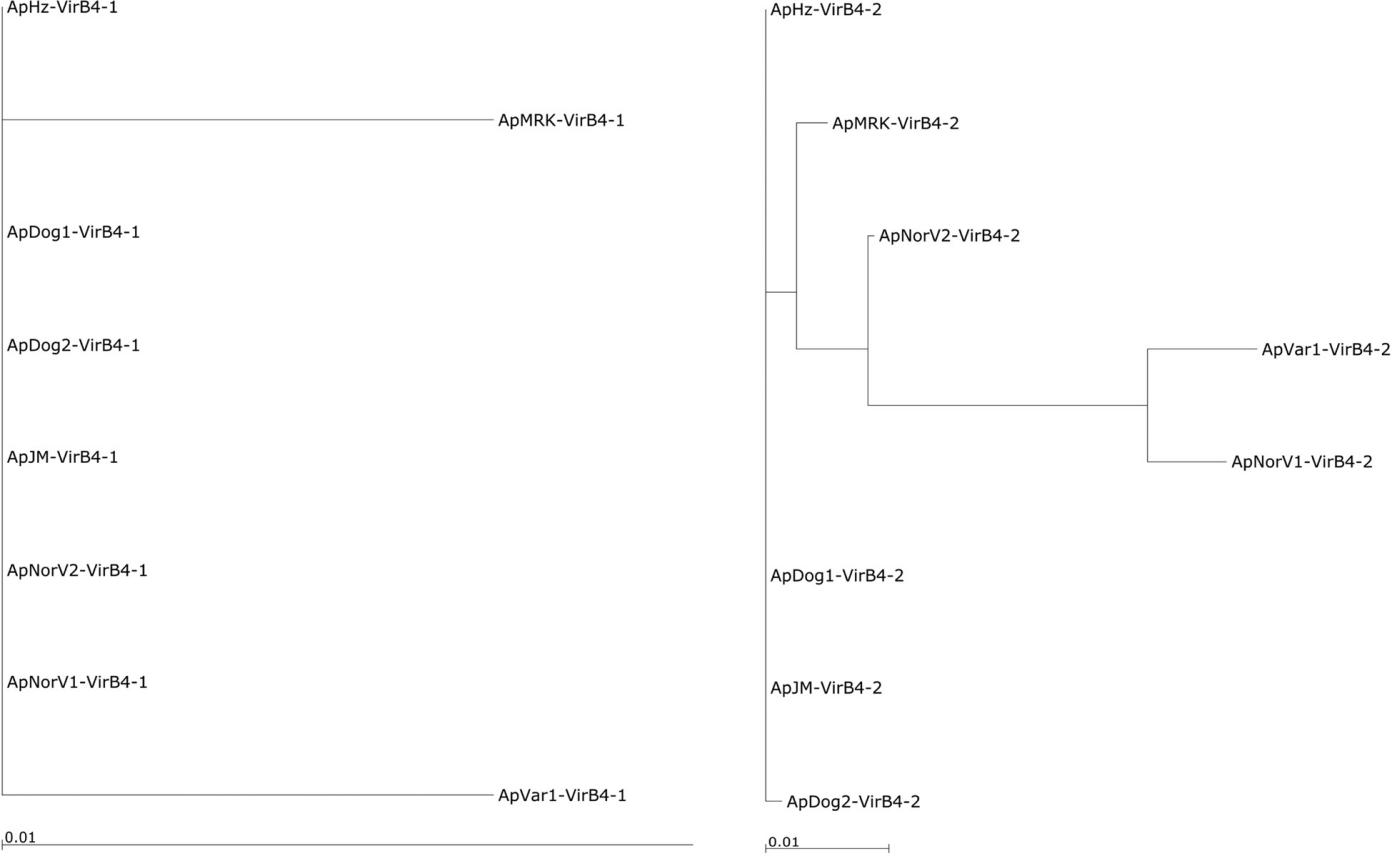
**Phylogenetic trees to show the relationship of syntenic VirB4 proteins from different strains of *A. phagocytophilum*.**

ATPases are typically used in T4SS to energize substrate transfer and have been found in every T4SS described. In gram-negative bacteria these are typically integral membrane proteins encoded by genes residing upstream of *virB2* (encoding pilin). This is true for all strains of *A*. *phagocytophilum* and it has been suggested that this arrangement of multiple *virB2* paralogs and *virB42* may allow assembly of an antigenically variable surface organelle [20]. The energetic subunit itself, VirB4-2, is however, well conserved between strains. The most distant taxonomic relationship was found between human and ruminant strains (29 total amino acid substitutions in *Ap*NorLamb-V1 compared to *Ap*HZ, Figure 7). The other energetic subunit, VirB11, was also well-conserved between strains (6 amino acid substitutions between *Ap*NorLamb-V1 and *Ap*HZ; data not shown).

### Type 4 coupling protein: VirD4

**Figure 8:**
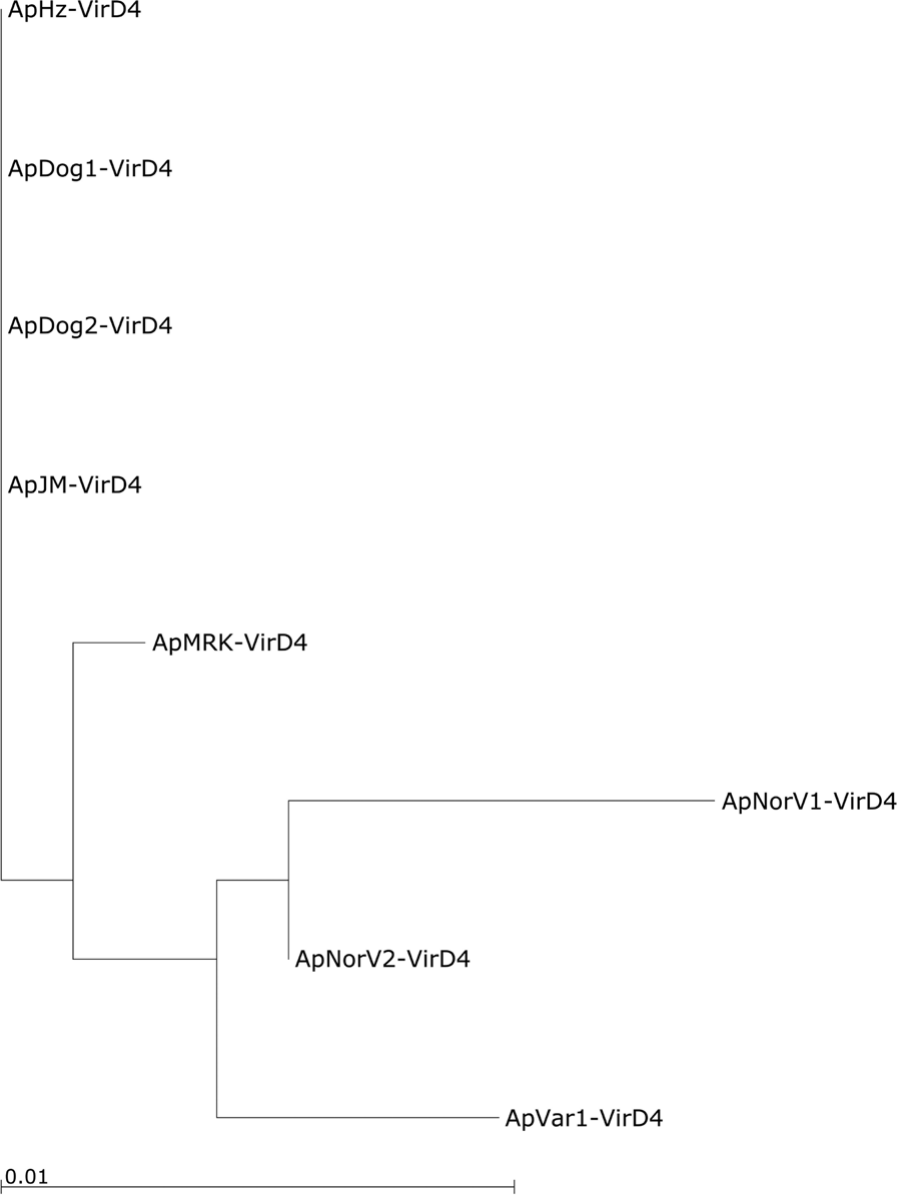
**Phylogenetic tree to show the relationship of syntenic VirD4 proteins from different strains of *A. phagocytophilum*.**

Type 4 coupling proteins such as VirD4 are ATPases that function in substrate recognition and translocation using the T4SS. They are associated with most effector translocator systems. They typically possess a minimum of two N-terminal transmembrane domains. Often most heterogeneity exists in these N-terminal regions [20]. The *A*. *phagocytophilum* VirD4^′^s conform somewhat to this stereotype with three strongly predicted N-terminal transmembrane segments. As with the other ATPases of the *A*. *phagocytophilum* T4SS, there is little variation in VirD4, a total of 17 amino acid substitutions of which 4 are N-terminal but more (12) are C-terminal. Again, the evolutionary relationships among VirD4 sequences position the ruminant and horse strains more distantly to the U.S. dog, human and rodent strains (Figure 8).

## Conclusions

**Figure 9:**
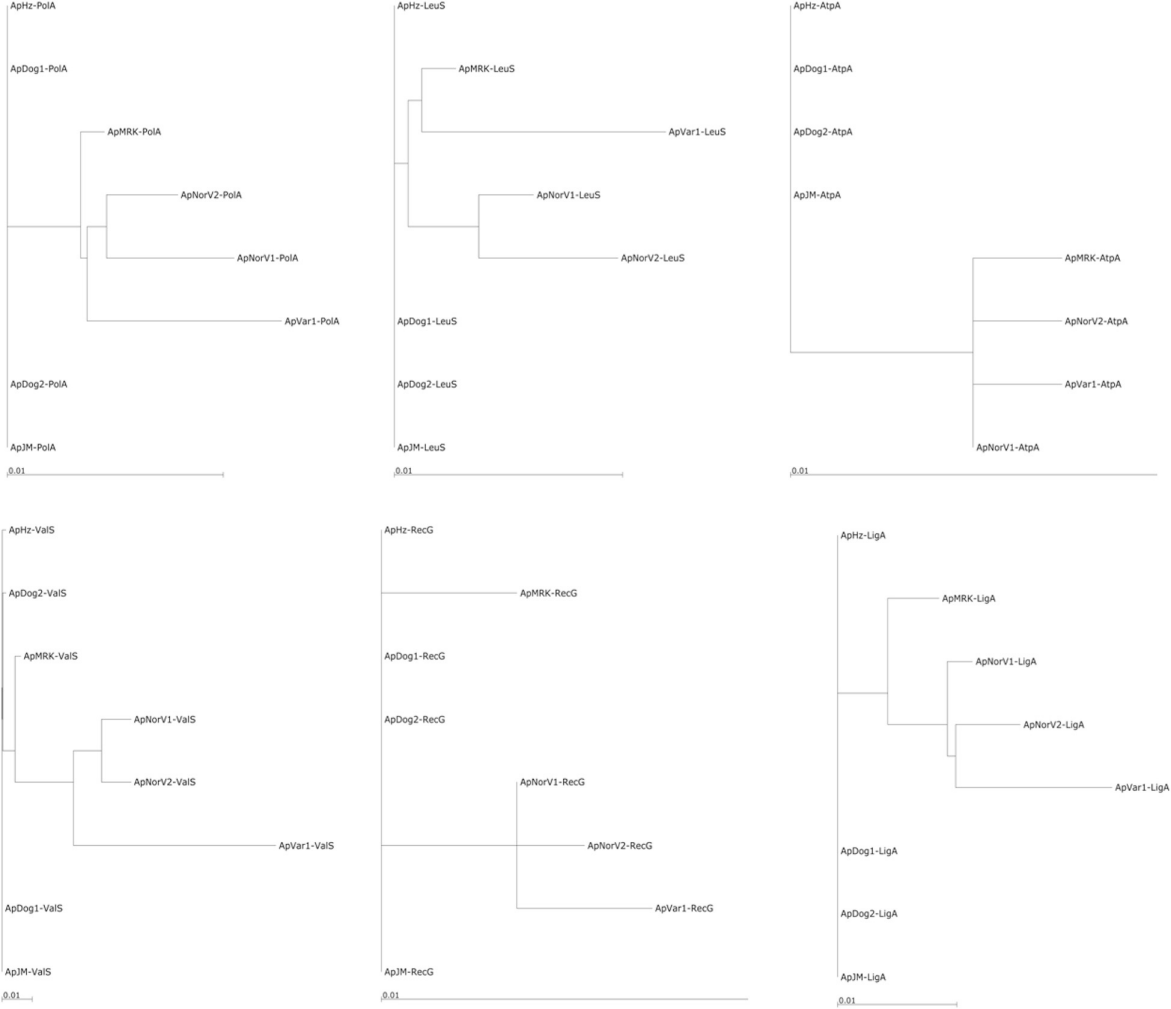
**Phylogenetic tree to show the relationship of other conserved proteins from different strains of *A. phagocytophilum. ***These proteins are: PolA, DNA polymerase I; LeuS, leucyl-tRNA synthetase; AtpA, ATPsynthase F1, alpha subunit; ValS,valyl-tRNA synthetase; RecG, ATP-dependent DNA helicase; LigA, NAD-dependent DNA ligase.

*A*. *phagocytophilum* represents a recent reclassification of intracellular organisms infecting different animal species and humans and causing diverse disease symptomatology [43]. These bacteria were previously known as *Ehrlichia phagocytophila*, *Ehrlichia equi*, and the agent of human granulocytic ehrlichiosis. Despite the differences within this species, the overall genome structure and synteny of the T4SS is maintained. However, gene structural analysis reveals evidence of gene duplication and considerable diversity of T4SS components in strains infecting different animals. Taxonomic trees suggest a close evolutionary relationship of *A*. *phagocytophilum* strains infecting U.S. humans, mice and dogs and a more distant relationship with ruminant and horse strains. This relationship is not unique to the T4SS but is also supported by similar taxonomic trees of other *A*. *phagocytophilum* proteins of conserved metabolic function (Figure 9). Within the T4SS multicomponent membrane complex, the energetic and internal scaffolding protein components are the most conserved. In contrast, components that form the proposed exposed structures of the T4SS, such as VirB2 and VirB6, are more variable. T4SS are important virulence determinants of bacteria, therefore these differences may result in the different infectivity and virulence profiles observed with different strains. It will be of interest to determine the molecular architecture of VirB6 paralogs in different strains, including interactions with other T4SS components and effectors. Of the known surface exposed components of the T4SS, VirB9 is the most conserved. This protein has been proposed as a vaccine component against *A*. *marginale* and may also be suitable against *A*. *phagocytophilum*.

## Methods

### *A. phagocytophilum* strains, cell culture, and experimental infection

The *A*. *phagocytophilum* U.S. strains HZ (human-origin, NY), MRK (horse-origin, CA), JM (rodent-origin, MN) and Dog1 (dog-origin, MN) were propagated in HL-60 cells in RPMI-1640 medium (Thermo Fisher Scientific, Inc., Waltham, MA) supplemented with final 10% heat-inactivated fetal bovine serum (Thermo Scientific) and 4 mM L-glutamine (Lonza, Rockland, ME), and in the absence of antibiotics. *Ap*HZ and *Ap*MRK have been described previously [15,44]. The *Ap*JM strain (CR01-1258) originated from a meadow jumping mouse (*Zapus hudsonius*) trapped at Camp Ripley, MN [45]. The *Ap*Dog1 strain originated from the blood of a dog from Baxter, MN naturally infected with *A*. *phagocytophilum*, as evidenced by the detection of distinctive morulae in a diagnostic blood sample, and sequencing of the Expression Site-linked *msp2*/*p44* gene. Briefly, whole blood was collected from the animal with EDTA as an anticoagulant. The buffy coat layer was collected after low-speed centrifugation of the whole-blood, washed in 1x phosphate buffered saline (PBS, Hyclone, cat. no. SH30256.01), then added to a culture of uninfected HL-60 cells. The culture was left undisturbed for 3 days, after which morulae began to appear. The *Ap*Dog2 strain also originated from a MN dog and was passaged to and maintained in the *Ixodes scapularis* ISE6 tick cell line as described [46]. The Ap variant 1 CRT35 strain (tick-origin, MN), maintained in ISE6 cells, has been described [47]. For DNA isolation, cultures were maintained until 90-100% of cells were infected with mature morulae. Cells were pelleted by centrifugation at 2500 x g for 20 min at 4°C. Pellets were gently resuspended in 1.5 ml cold PBS, transferred to screw-cap microfuge tubes, and centrifuged at 1500 x g for 20 min at 4°C. Supernatants were removed and the cell pellets stored at −80°C until further use.

Two naturally occurring Norwegian lamb *A*. *phagocytophilum* strains differing in the *16S* rRNA gene and degree of virulence were used to experimentally infect lambs raised in an indoor environment with barriers against tick entry and tick infestation. Lamb 00186 was infected with the more virulent variant 1 (identical to GenBank M73220) and lamb 0054 with variant 2 (identical to GenBank AF336220) [48], to be referred to as *Ap*NorLamb-V1 and -V2 from here on. Infections were monitored by microscopy and blood was harvested at maximum parasitemia. To purify buffy coats containing the infected neutrophils, approximately 2.5 l of Na-citrated blood was collected from each animal. The blood was transferred to 1 l centrifuge bottles and centrifuged at 2,500-3,000 x g in a swing-out bucket rotor for 30 min at 4°C. After removing most of the plasma layer, the buffy coat layer was collected with minimal contamination of red blood cells. The cells were diluted 1:3 with PBS, mixed gently and centrifuged at 1,500x g for 20 min at 4°C. Following three PBS washes, supernatants were removed and the cell pellets stored at −80°C. The experimental study in sheep was approved by the Norwegian Animal Research Authority.

### Purification of host cell-free *A*. *phagocytophilum* and genomic DNA (gDNA) isolation

For the HZ, JM, Dog1, MRK and NorLamb-V1 and -V2 strains, intact, host cell-free organisms with minimal host cell gDNA/RNA contamination were purified from frozen PBS pellets of infected cells prepared as above. Samples and reagents were maintained on ice throughout the entire procedure, and all centrifugations performed at 4°C. Following a quick thaw, host cells were disrupted by vigorous vortexing for 5 min. An equal volume of PBS was added and vortexing continued for 3 min. Cellular debris was removed by centrifugation at 200 x g for 15 min. After removing most of the supernatants to fresh tubes, these were passed several times through a 31 G needle and saved on ice. Pellets were resuspended well in final 500 μl PBS then passed serially through 22 G, 25 G, 28 G and, when possible, 31 G needles attached to a 1 ml syringe. 3–5 volumes PBS were added and mixed by vortexing. Debris was removed by centrifugation at 200 x g for 10 min. Supernatants were pooled to those from the previous centrifugation step. RNaseA was added to a final 250–300 μg/ml and the samples incubated 45–60 min at 37°C. Samples were centrifuged at 21,000 x g for 30 min and the supernatants removed completely. Pellets were resuspended in 50–100 μl PBS each and transferred to fresh tubes. To ensure homogeneity of the suspension, initially a drawn-out 10 μl pipette tip was used to disrupt the pellet by swirling followed by up/down pipetting and gentle vortexing, before switching to a larger tip. The sample was further homogenized by several passes through a 28-31 G needle. PBS was added to final 500–700 μl and DNaseI to final 250 μg/ml. Following 45–60 min incubation at 37°C the samples were centrifuged at 21,000 x g for 30 min. Pellets were homogenized as above and the DNaseI treatment repeated. EDTA (pH 8.0) was added to final 25 mM and the samples centrifuged as above. Tubes were washed twice with PBS without disturbing the pellets and residual PBS was removed after 3 min centrifugation at 21,000 x g. Pellets were homogenized as above in 600–800 μl RPMI culture medium (containing 10% fetal bovine serum) added incrementally and transferred to a 50 ml tube. Culture medium was added to a final volume of 6 ml before passage through a pre-wet, 2 μm pore-size, 25 mm, GMF-150 glass microfiber syringe filter (Puradisc 25GD; Whatman Inc., Florham Park, NJ). The filter was washed 3-4x with culture medium. Washes were pooled to the filtrate and centrifuged at 22,000 x g for 30 min. The pellets, comprised of free, non-viable organisms and host cell mitochondria, were resuspended in PBS, transferred to microfuge tubes and re-pelleted at 21,000 x g for 30 min. Supernatants were removed completely and the pellets were processed immediately or stored at −20°C. For every 10^8^ host cells used at 90-100% infectivity, enough organisms were recovered to yield on average 1–1.5 μg high-quality DNA using either the Gentra Puregene Yeast/Bact. kit (Qiagen Inc., Valencia, CA) or the QIAGEN Blood & Cell Culture DNA mini kit following the manufacturer’s protocols.

For the Dog2 and Ap variant 1 strains, organisms were cultured and isolated from ISE6 tick cells as described [49]. Host cell-free bacteria were prepared from two cultures in 25 cm^2^ flasks, collected by centrifugation for 10 min at 11,000 xg at 4°C, and lysed in Gentra Puregene lysis buffer (Qiagen) at 80°C for 5 min. Since these DNA samples also contained a considerable amount of small (<500 bp) DNA species naturally associated with the ISE6 host cell line, the *A*. *phagocytophilum* gDNA was further purified by electroelution from agarose gels, followed by phenol/chloroform extraction and EtOH precipitation using conventional protocols.

### Preparation of host cell-free *A*. *phagocytophilum* agarose plugs for optical mapping

*Ap*Dog1 was initially selected for complete genome sequencing to compare with the published HZ strain. When a draft genome was assembled for *Ap*Dog1 it was largely syntenic with HZ except for the *vir*B6 locus, indicating a possible error in the sequence of one or both of the strains. Accordingly, the *Ap*Dog1 draft genome sequence was verified by Optical Mapping. In preparation for Optical Mapping (performed by OpGen Inc., Gaithersburg, MD), host cell-free organisms were embedded in 0.5% low-melting point agarose plugs and subsequently lysed, allowing access to the intact, ~1.48 Mb circular *A*. *phagocytophilum* chromosome. A procedure recommended by OpGen was followed. All solutions were made fresh using OpGen suggested reagents. Intact *Ap*Dog1 organisms were purified as above, except that the pellet of free organisms obtained following centrifugation of the filtrate was resupended and washed in cell suspension buffer [200 mM NaCl, 100 mM EDTA-Na_2_ (pH 8.0), 10 mM Tris (pH 7.2)]. Plugs were made immediately on completion of the isolation procedure. Briefly, following the final centrifugation of the purified organisms, the pellet was resuspended in cell suspension buffer using 40–50 μl for every 10^8^ host cells used at >95% infectivity. The sample was passed 2x through a 31 G needle (3/10 ml capacity Insulin Syringe with fused 8 mm long needle, BD #328438; Becton, Dickinson & Co., Franklin Lakes, NJ) to ensure homogeneity of the thick suspension, and an equal volume of 1% low melting point SeaPlaque GTG agarose [(Lonza #50111) dissolved in DEPC-treated water (Invitrogen #750023; Carlsbad, CA) and held at 55°C] was immediately added. Following mixing, 100 μl aliquots were dispensed into plug molds (Bio-Rad #170-3713; Hercules, CA) and allowed to set for 1 hr at 4°C prior to transfer into a 50 ml tube containing 5–10 ml, 50°C NDSK solution [filter sterile NDS solution (1% N-lauroylsarcosine (Sigma #L5000; St. Louis, MO) in 0.5 M EDTA-Na_2_ (pH 9.5), supplemented with final 2 mg/ml proteinase K (Pierce #17916; Rockford, IL) immediately prior to use]. The tube was incubated upright at 50°C with mild shaking (40 rpm) for 8–24 hrs, until the plugs turned clear and colorless. Plugs were gently washed 3x in 5 ml 0.5 M EDTA-Na_2_ (pH 9.5), then transferred to a fresh tube and stored in EDTA at 4°C. Optical Mapping data generated from the BamHI-digested *Ap*Dog1 chromosome was analyzed using the OpGen MapSolver software.

### 454 Genome sequencing and bioinformatics

Isolated DNA was provided to the Interdisciplinary Center for Biotechnology Research (ICBR) core facilities, University of Florida for library construction and pyrosequencing on the Roche/454 Genome Sequencer according to standard manufacturer protocols. Regular read libraries were generated for all strains. Additionally, 3 kb paired end libraries were made for *Ap*HZ, *Ap*Dog1 and *Ap*MRK. Genome coverage range was 31.3x to 72.1x. For each strain, the SFF format flow files, returned by ICBR for bioinformatics analysis, were first combined and converted to .fasta and .qual files (or the two combined in .fastq format) using Roche/454 Genome Sequencer FLX System software. Genome drafts were assembled using the CLC Genomics Workbench software suite (version 4.0-4.9) by mapping reads initially against the fully annotated, Sanger sequenced *Ap*HZ genome (GenBank CP000235), then against the completed *Ap*Dog1 genome. Default parameters were used: length fraction, 0.5; similarity, 0.8; and for paired end reads, minimum distance, 1500/maximum distance, 4500. To obtain the *vir* loci, the resulting consensus sequence and underlying aligned reads were inspected for conflicts and mismatched paired ends suggesting the presence of insertions and/or deletions not mirrored in the consensus. These were manually corrected. Gaps were also manually closed where possible. Briefly, overlapping reads covering at least 2 kb of sequence on both sides of a gap and extending into it were individually extracted from the alignment. A new consensus for each side was obtained by assembling the reads against each other, and 250 N’s were added to its ends. These were individually used as the reference sequence against which all the 454 reads were re-mapped to pull out novel reads extending into the unknown region. The process was repeated multiple times, allowing for the incremental filling of the gap. PCR, followed by sequencing was performed when sequences extrapolated in this fashion spanned complex tandem repeat regions such as repeat regions 1 and 3 (R1 and R3 in Figure 3A) of the *virB6*-*4* gene, or when gap closure could not be completed due to such structures, as was the case with the extremely long *virB6*-*4* R4 (Figure 3A) region.

Amino acid sequences were aligned with MAFFT [50] and displayed with CHROMA [51]. Taxonomic relationships used a neighbor-joining tree and the ITT substitution model [52] and were displayed using Archaeopteryx (http://www.phylosoft.org/archaeopteryx). Hydrophobicity analyses were conducted using the method of Hopp and Woods [53,54] at web.expasy.org and transmembrane segments were predicted with TMpred at http://www.ch.embnet.org/software/TMPRED_form.html.

### PCR amplification of virB6-4 gene repeat regions, cloning, and Pacific Biosciences sequencing

Due to difficulties in amplifying tandem repeat-containing DNA, all PCR reactions spanning the *virB6*-*4* gene repeat regions were performed in the presence of 1.5-1.7 M Betaine (Sigma). The 8.36 kb PCR product spanning R3 and R4 in the *Ap*HZ strain (Figure 3A, 3C, and Additional file 2: Figure S2A) was amplified using the iProof High-Fidelity DNA Polymerase system with GC buffer (Bio-Rad). Reactions totaled 50 μl with 5 ng purified *A*. *phagocytophilum* gDNA, 1.0 U polymerase, 1.5 mM MgCl_2_, 200 μM each dNTP, and 250 nM each primer (AB1393: 5^′^-CGGGATCTAAGACAGATGATGATTC-3^′^, forward; AB1466: 5^′^-CTCATCCTGATGCGTCTCCTTAG-3^′^, reverse; Figure 3A). 35 cycles of 30 sec denaturing at 98°C, 20 sec annealing at 67°C, and 5 min extension at 72°C were performed. PCR products spanning R4 in *Ap*JM and *Ap*Dog1 (both ~10.3 kb; Figure 3C) were derived using Takara’s PrimeSTAR GXL DNA Polymerase system (Clontech Laboratories, Mountain View, CA). Reactions contained 5 ng gDNA, 1.25 U polymerase, 1.0 mM MgCl_2_, 200 μM each dNTP, and 200 nM each primer (AB1395: 5^′^-CACCAGAGGATGCAGCATTAG-3^′^, forward; AB1466, reverse; Figure 3A) in total 50 μl. Following the manufacturer’s recommendations, 2-step PCR was performed with 30 cycles of 10 sec denaturing at 98°C and 10 min annealing/extension at 68°C. PCR products were analyzed on 0.5% agarose gels alongside the 1 kb Plus (Invitrogen) and the GeneRulerHighRange (Fermentas, Inc., Glen Burnie, MD) DNA ladders. In order to TA-clone the amplicons, A-overhangs were added to the ends using 0.5-1.0 units AmpliTaq DNA polymerase (Applied Biosystems, Foster City, CA) in a 10–15 min reaction at 72°C. Products purified from agarose gels (before or after A-overhang addition) were cloned into the pCR-XL-TOPO vector (Invitrogen) and transformed into *E*. *coli* Stbl2 (Invitrogen), which is more permissive to repeat-laden foreign DNA. Recombinants containing the correct size insert were end sequenced to verify their identity.

In preparation for sequencing with the long-read length Pacific Biosciences (PacBio) next-generation sequencing RS instrument, constructs were linearized with restriction enzymes which cut the vector only, but on opposite sides of the insert within the Multiple Cloning Site. For *Ap*HZ, equimolar amounts of the TA clone were cut with either HindIII or EcoRV. Following pooling and EtOH precipitation, the linearized DNA mix was submitted to ICBR/UF for SMRTbell library construction and sequencing. Libraries were constructed using a commercial strobe library preparation kit (#001-326-530; Pacific Biosciences, Menlo Park, CA) following standard manufacturer protocols. To further increase the likelihood of full coverage, the strobe-sequencing run was performed using two different conditions: I) 45 min light period (continuous collection time); and II) (5 min light period, 10 min dark period), followed by (45 min light period, 10 min dark period). The *Ap*JM and *Ap*Dog1 constructs were double-digested with HindIII/XbaI to excise the ~10.3 kb inserts. Following separation on 0.5% agarose gels, the inserts were recovered from agarose slices by electroelution and further purified and concentrated by passage over QIAquick spin columns following the PCR Purification kit protocol (Qiagen). SMRTbell libraries were made as above then sequenced using a single 75 min movie time run.

Due to the repetitive nature of the cloned gene fragments, combined with the relatively high error-rate of the PacBio system, all attempts to assemble the reads *de novo* failed to yield a sequence of the expected size. Therefore, for each construct, reads >3 kb were selected from the multi-fasta files using the Galaxy suite [55], and imported into the CLC Genomics Workbench for assembly and further analysis. These were assembled at low stringency initially against a consensus sequence representing an entire (vector and insert sequence) linear construct to which sufficient N’s were added based on the estimated gap-size. Starting with reads initiating outside the repeat region, the longest of the assembled reads were visually inspected for the presence of *virB64* R4 repeat signature-sequences (Additional file 2: Figure S2) and their sequence manually corrected where necessary. The extended sequences were used to replace N’s in the consensus and the process repeated several times until sufficient reads with >2 kb sequence overlap were recovered spanning the entire insert region. For verification, the completed sequence for each strain was used as the reference to re-map all the respective >3 kb PacBio reads and the Roche/454 reads at higher stringency.

### GenBank Accession Numbers: for each isolate, the *vir* genes are listed in order

The sequences of *vir* loci are complete for strains *Ap*Dog1 and *Ap*JM. The sequence of the repetitive *virB64* locus was incomplete (*Ap*Dog2) or not determined for the other strains except *Ap*Hz. We provide a revised sequence of *virB64* for the previously sequenced [15]*Ap*HZ strain.

*Ap*Dog1:JX415845 - JX415868

B2-1 B2-2, B2-3, B2-4, B2-5, B2-6, B2-7, B2-8, B2-9, B3, B4-1, B4-t1, B4-2, B6-1, B6-2, B6-3, B6-4, B8-1, B8-2, B9-1, B9-2, B10, B11, D4

*Ap*JM:JX415869 - JX415892

B2-1, B2-2, B2-3, B2-4, B2-5, B2-6, B2-7, B2-8, B2-9, B3, B4-1, B4-t1, B4-2, B6-1, B6-2, B6-3, B6-4, B8-1, B8-2, B9-1, B9-2, B10, B11, D4

*Ap*Dog2:JX415893 - JX415915 (*virB6-4* submitted separately as gapped)

B2-1, B2-2, B2-3, B2-4, B2-5, B2-6, B2-7, B2-8, B2-9, B3, B4-1, B4-t1, B4-2, B6-1, B6-2, B6-3, B8-1, B8-2, B9-1, B9-2, B10, B11, D4

*Ap*NorLambV2:JX415916 - JX415938

B2-1, B2-2, B2-3, B2-4, B2-5, B2-6, B2-7, B2-8, B2-9, B3, B4-1, B4-t1, B4-2, B6-1, B6-2, B6-3, B8-1, B8-2, B9-1, B9-2, B10, B11, D4

*Ap*NorLambV1:JX415939 - JX415966

B2-1, B2-2, B2-3, B2-4, B2-5, B2-6, B2-7, B2-8, B2-novel1, B2- novel2, B2-novel3, B2-novel4, B2-novel5, B2-novel6, B3, B4-1, B4- t1, B4-2, B6-1, B6-2, B6-3, B8-1, B8-2, B9-1, B9-2, B10, B11, D4

*Ap*HZ*virB6-4*:JX415967

*Ap*Var1:JX415968 - JX415996

B2-1, B2-2, B2-3, B2-4, B2-5, B2-6, B2-7, B2-8, B2-novel1, B2- novel2, B2-novel3, B2-novel4, B2-novel5, B2-novel6, B2-novel7, B3, B4-1, B4-t1, B4-2, B6-1, B6-2, B6-3, B8-1, B8-2, B9-1, B9-2, B10, B11, D4

*Ap*MRK:JX415997 - JX416019

B2-1, B2-2, B2-3, B2-4, B2-5, B2-6, B2-7, B2-8, B2-9, B3, B4-1, B4-t1, B4-2, B6-1, B6-2, B6-3, B8-1, B8-2, B9-1, B9-2, B10, B11, D4

ApDog2*virB6-4*Gapped:JX416020.

## Competing interests

The authors declare that they have no competing interests.

## Authors’ contributions

BAK and AFB conceived the study, performed bioinformatics analyses and drafted the manuscript. BAK grew infected HL-60 cell cultures, purified organisms, isolated gDNA, designed and supervised PCR and submitted sequences to GenBank. AML performed PCR analyses and cloning and supervised data transfer between units. SS and EGG isolated the European sheep strains, infected and monitored sheep, and prepared organisms at maximal parasitemia. UGM and CMN isolated and cultured *in vitro* the JM, MRK, Dog2 and Ap variant 1 strains, and prepared Dog2 and Ap variant 1 strain gDNA. ARA and SMM established the Dog1 strain. All authors read and approved the final manuscript.

## Supplementary Material

Additional file 1 **Figure S1.** Multiple sequence alignment of VirB6-3 amino acid sequences from different strains of A. phagocytophilum. Arrows indicate the locations of C-terminal 41-mer repeats. Click here for file

Additional file 2 ** Figure S2.** Structure of the virB6-4 repeat regions R3 and R4 in four US A.phagocytophilum strains.A. Comparative maps of AB1393/AB1466 PCR products detailing the repeat unit content of R3 and R4 in the human, rodent and dog strains. *Ap*JM and *Ap*Dog1 have identical *virB6*-*4* genes and are, therefore, represented by one map. Moderate variability in the number and sequence of the R3 405 bp repeat units (light blue arrows) is apparent. The small bar at the end of R3 corresponds to the 3^′^-most partial repeat unit present in all strains. The colored arrows within R4 represent the five repeat types T1a (yellow), T1b (green), T2a, (red), T2b (dark blue) and T2c (grey). The repeat pattern in *Ap*HZ shows no relationship to that of *Ap*JM/*Ap*Dog1, which is also 2.87 kb larger, totaling 9.76 kb. This region was not fully characterized in *Ap*Dog2 as indicated by a broken line, but the repeat pattern of the 5^′^- and 3^′^-most repeats is clearly different from that of the other strains. The small bar downstream of the second repeat unit represents a partially characterized type 2 repeat unit. Lines above and below the *Ap*HZ and *Ap*JM/*Ap*Dog1 maps delineate segments of sequence identity within the respective R4 regions. Their sizes are specified. **B**. Alignment of the nucleic acid sequence of all *virB6*-*4* R4 repeat unit types identified to date. Type 1 repeats are shown in black, type 2 in blue. Differences between sub-types are highlighted. A single BamHI site present in all type 2 repeats is underlined. With the exception of only a few nucleotides at each end, type 1 and type 2 repeat units do not share any sequences. **C**. Alignment of the amino acid sequences of the repeat units shown in B. The single nucleotide differences between sub-types do not lead to changes in amino acid sequence. Click here for file
