# Supplementary material for: Structure of the type IV secretion system in different strains of Anaplasma phagocytophilum
Source: BMC Genomics. 2012 Nov 29;13:678. doi: 10.1186/1471-2164-13-678 (PMC3556328; doi:10.1186/1471-2164-13-678)
Supplement: Additional file 1 — Figure S1. Multiple sequence alignment of VirB6-3 amino acid sequences from different strains of A. phagocytophilum. Arrows indicate the locations of C-terminal 41-mer repeats. [file 1471-2164-13-678-S1.pdf]

Fig. S1

```
ApHZ-VirB6-3       ML-SRSLLFIAIVVLSSCGGTCIEPGAGVSSSSQEVQVPVYPDGADHKRPVTYWVHSGYRVGEKDELKITVDRTIDLCPLDTKAKPVAIKMYPEHFST--
ApDog1-VirB6-3     ML-SRSLLFIAIVVLSSCGGTCIEPGAGVSSSSQEVQVPVYPDGADHKRPVTYWVHSGYRVGEKDELKITVDRTIDLCPLDTKAKPVAIKMYPEHFST--
ApDog2-VirB6-3     ML-SRSLLFIAIVVLSSCGGTCIEPGAGVSSSSQEVQVPVYPDGADHKRPVTYWVHSGYRVGEKDELKITVDRTIDLCPLDTKAKPVAIKMYPEHFST--
ApJM-VirB6-3       ML-SRSLLFIAIVVLSSCGGTCIEPGAGVSSSSQEVQVPVYPDGADHKRPVTYWVHSGYRVGEKDELKITVDRTIDLCPLDTKAKPVAIKMYPEHFST--
ApMRK-VirB6-3      ML-SRSLLFIAIVVLSSCGGTCIEPGAGVSSSSQEVQVPVYPDGADHKRPVTYWVHSGYRVGEKDELKITVDRTIDLCPLDTKAKPVAIKMYPEHFST--
ApNorV2-VirB6-3    MLLRRLLLFIAIVVLSSCGGTCIEPGAGVSSSSQEVQVPVYPDGADHNRPVTYWVHSGYRVGEKDELKITVDRTIDLCPLDTKAKPVAIKMYPEHFST--
ApVar1-VirB6-3     MLL-RLFLFIAIVVLSSCGGTCIEPGAGVSSSSQEVQVPVYPDGADQKRPVTYWVNSGHRVGEKDELKITVDRTIDLCPLDTKAKPAVIRMYPEHFST--
ApNorV1-VirB6-3    MML-RLLLVIAIVALSSCGGTCIEPGAGVSSSSQEVQVPVYPDGADQKRPVTYWVHSGHRVGEKDEIKITVDRTIDLCPLDTKAKPAVIRMYPEHLSTSI
Consensus/80%      ML..R.LLFIAIVVLSSCGGTCIEPGAGVSSSSQEVQVPVYPDGADpKRPVTYWVHSGaRVGEKDELKITVDRTIDLCPLDTKAKPssI+MYPEHFST..

ApHZ-VirB6-3       -PSTEFYDTYIDVQEGDQLRFSSVLYDLSFPDCKEISREHSPVIFHNNEVIFRDEACKKTVAIEELCLSGATSEVLGDGSKKGLLHVKDAKGECKEVPAG
ApDog1-VirB6-3     -PSTEFYDTYIDVQEGDQLRFSSVLYDLSFPDCKEISREHSPVIFHNNEVIFRDEACKKTVAIEELCLSGATSEVLGDGSKKGLLHVKDAKGECKEVPAG
ApDog2-VirB6-3     -PSTEFYDTYIDVQEGDQLRFSSVLYDLSFPDCKEISREHSPVIFHNNEVIFRDEACKKTVAIEELCLSGATSEVLGDGSKKGLLHVKDAKGECKEVPAG
ApJM-VirB6-3       -PSTEFYDTYIDVQEGDQLRFSSVLYDLSFPDCKEISREHSPVIFHNNEVIFRDEACKKTVAIEELCLSGATSEVLGDGSKKGLLHVKDAKGECKEVPAG
ApMRK-VirB6-3      -PSTEFYDTYIDVQEGDQLRFSSVLYDLSFPDCKEISSEHSPVIFHNNEVIFRDEACKKTVAIEELCLSGATSEVLGDGSKKGLLHVKDAKGECKELPAG
ApNorV2-VirB6-3    -PSTEFYDTYIDVQEGDQLRFSSVLYDLSFPDCKEISSEHSPVIFHNNEVIFQDSSCKKAVEIEELCLSGATSEVLGDGSKKGLLHVKDAKGNCKELPAG
ApVar1-VirB6-3     -PSTEFYDTYIDVQEGDQLRFGAVLYDLNFPNCKEISSEHSPVIYHNNEVIFQDSSCKKAVEAEELCLSGATSEVLGDGSKKGLLYVKDAGGECKELPAG
ApNorV1-VirB6-3    EPSTEFYDTHIDVQEGDQLRFGAVLYDLNFPNCKEISSEHSPVIYHNNEVIFRDGACKKAVEAEELCLSGALSEKVGE--KKGLLYVKDSKGECQALPES
Consensus/80%      .PSTEFYDTYIDVQEGDQLRFttVLYDLsFPsCKEISpEHSPVIaHNNEVIFpDptCKKsV.hEELCLSGATSEVLGDGSKKGLLaVKDAKGECKElPAG

ApHZ-VirB6-3       VQIPRVGGSIIQTPRMQFPLGYIGVVDSSVSGELSFSYNNVITNGRIHDARVPKINMSPKLDQESCELLKGSNITLRLSNIDSIDQLKDRDAHNKHERSY
ApDog1-VirB6-3     VQIPRVGGSIIQTPRMQFPLGYIGVVDSSVSGELSFSYNNVITNGRIHDARVPKINMSPKLDQESCELLKGSNITLRLSNIDSIDQLKDRDAHNKHERSY
ApDog2-VirB6-3     VQIPRVGGSIIQTPRMQFPLGYIGVVDSSVSGELSFSYNNVITNGRIHDARVPKINMSPKLDQESCELLKGSNITLRLSNIDSIDQLKDRDAHNKHERSY
ApJM-VirB6-3       VQIPRVGGSIIQTPRMQFPLGYIGVVDSSVSGELSFSYNNVITNGRIHDARVPKINMSPKLDQESCELLKGSNITLRLSNIDSIDQLKDRDAHNKHERSY
ApMRK-VirB6-3      VQIPRVGGSIIQTPRMQFPLGYIGVVDSSVSGELSFSYNNVITNGRIHDARVPKINMSPKLDHESCELLKGSNITLRLSNIDSVDQLKDRDAHNKHERSY
ApNorV2-VirB6-3    VQIPRVGGSIIQTPRMQFPLGYIGVVDSSVSGELSFSYNNVITNGRIHDARVPKINMSPKLDEESCALLKESNITLRLSNIDSVDQLKDRDAHNKHERSY
ApVar1-VirB6-3     VQIPRVGGSIIQTPRMQFPLGYIGVVDSSVSGELSFSYNNVITNGRIHDARVPKMNMSPKLDQESCELLKGSNLTLRVSNIDSVDQLKDRDAHNKHERSY
ApNorV1-VirB6-3    VQIPRVGGSIIQTSRMQYPLGYIGVVDSSVSGELSFSYNNVITNGRIHDARVPKMNASPKLDQESCELLKGSNLTLRVSNIDSVDQLKDRDAHNKHKRSY
Consensus/80%      VQIPRVGGSIIQTPRMQFPLGYIGVVDSSVSGELSFSYNNVITNGRIHDARVPKbNMSPKLDpESCELLKGSNlTLRlSNIDSlDQLKDRDAHNKHERSY

ApHZ-VirB6-3       TYGSNKAFTTGKRFGKPSETEEEKKQREKEEKIAALLSEYTEYDLNCHCGYVCKPSNQVDDDCIRSIVTVMDGNVVCPTQTKFNGKDHEEDAGNQQHNIP
ApDog1-VirB6-3     TYGSNKAFTTGKRFGKPSETEEEKKQREKEEKIAALLSEYTEYDLNCHCGYVCKPSNQVDDDCIRSIVTVMDGNVVCPTQTKFNGKDHEEDAGNQQHNIP
ApDog2-VirB6-3     TYGSNKAFTTGKRFGKPSETEEEKKQREKEEKIAALLSEYTEYDLNCHCGYVCKPSNQVDDDCIRSIVTVMDGNVVCPTQTKFNGKDHEEDAGNQQHNIP
ApJM-VirB6-3       TYGSNKAFTTGKRFGKPSETEEEKKQREKEEKIAALLSEYTEYDLNCHCGYVCKPSNQVDDDCIRSIVTVMDGNVVCPTQTKFNGKDHEEDAGNQQHNIP
ApMRK-VirB6-3      TYGSNKAFTTGKRFSKPSETEEEKKQREKEEKIAALLSEYTEYDLNCHCGYVCKPSNQVDDDCIRSIVTVMDGNVVCPTQTKFNGKDHEEDAGNQQHNIP
ApNorV2-VirB6-3    TYGSNKAFTTGKRFSKPLETEEEKKQREKEEKIAALLSEYTEYDLNCHCGYVCKPSNQVDDDCIRSIVTVMDGNVVCPTQTKFNGKDHEEDAGNQQHNIP
ApVar1-VirB6-3     TYGSNKPFTTGKRFSKPSETEQEKKQREKEEKIAALLSEYTEYDLNCHCGYVCKPSNHVDDDCIRSIVTVMDGNVVCPTQTKFNGKDDEKDA-SQQHNIP
ApNorV1-VirB6-3    TDGDSKPFTTGKRFKKQLETKEEKEQREKEEKIAALLSEYTEYDLNCHCGYICKPSDKIDGSCVRSIVTVMDGNVVCPTQTKFNSKDDEEGAIKQQRNIP
Consensus/80%      TYGSNKsFTTGKRFtKP.ETEEEKKQREKEEKIAALLSEYTEYDLNCHCGYVCKPSNpVDDDCIRSIVTVMDGNVVCPTQTKFNGKDcEEDAhsQQHNIP
```

cont. Fig. S1

```
ApHZ-VirB6-3       DIRGDLSPEAFARSVSSLTGQHFGQN-VAQKDSVISVEKAYELAEGVIAVIVSIENGKKILPSDIAQYCNDHQGKCKYLPEGIKSLKSSQSGLTFEKGSL
ApDog1-VirB6-3     DIRGDLSPEAFARSVSSLTGQHFGQN-VAQKDSVISVEKAYELAEGVIAVIVSIENGKKILPSDIAQYCNDHQGKCKYLPEGIKSLKSSQSGLTFEKGSL
ApDog2-VirB6-3     DIRGDLSPEAFARSVSSLTGQHFGQN-VAQKDSVISVEKAYELAEGVIAVIVSIENGKKILPSDIAQYCNDHQGKCKYLPEGIKSLKSSQSGLTFEKGSL
ApJM-VirB6-3       DIRGDLSPEAFARSVSSLTGQHFGQN-VAQKDSVISVEKAYELAEGVIAVIVSIENGKKILPSDIAQYCNDHQGKCKYLPEGIKSLKSSQSGLTFEKGSL
ApMRK-VirB6-3      DIRGDLSPEAFARSVSSLTGQHFGQN-VAQKDSVISVEKAYELAEGVIAVIVSIENGKKILPSDIAQYCNDHQGKCKYLPEGIKSLKSSQSGLTFEKGSL
ApNorV2-VirB6-3    DIRGDLSPEAFARSVSSLTGQRFGQNDVAQNDSVISVEKAYELAEGVIAVIVSIENGKKIPEGEVAKYCNDHQEKCKYLPEGIKSLKSSQSGLTFEKGSL
ApVar1-VirB6-3     DIR-KMSQKDFASSVASLA----GQN-VLPKDSTISVKTAYELAEGVIAVIVSVENGKKIPEGGVAKYCNDHQGKCKYLPEGIKSLKVGQSGLTFEKGSL
ApNorV1-VirB6-3    DVY-NMSPEDMKKGLGALT----GQN-VLPKDSTIGVKTAYELAEGVIAVIVSVENGKKIPEGGVAEYCNDHQEKCKYLPDGIKSLKDGQSGLTFGDGSL
Consensus/80%      DIR.cbSPEsFA+SVtSLT....GQN.Vh.KDSsISVcpAYELAEGVIAVIVSlENGKKI..tslApYCNDHQ.KCKYLPEGIKSLKstQSGLTFEKGSL

ApHZ-VirB6-3       KLDSDYIAPGSGRLYLAYWPYFGELGKKVKERKGSEAAVVAASGSSSQRGLTVSRAKNYSENSLLSALAQRRFFWKQGMSSGSGGFLSSNDAGNTYVPLS
ApDog1-VirB6-3     KLDSDYIAPGSGRLYLAYWPYFGELGKKVKERKGSEAAVVAASGSSSQRGLTVSRAKNYSENSLLSALAQRRFFWKQGMSSGSGGFLSSNDAGNTYVPLS
ApDog2-VirB6-3     KLDSDYIAPGSGRLYLAYWPYFGELGKKVKERKGSEAAVVAASGSSSQRGLTVSRAKNYSENSLLSALAQRRFFWKQGMSSGSGGFLSSNDAGNTYVPLS
ApJM-VirB6-3       KLDSDYIAPGSGRLYLAYWPYFGELGKKVKERKGSEAAVVAASGSSSQRGLTVSRAKNYSENSLLSALAQRRFFWKQGMSSGSGGFLSSNDAGNTYVPLS
ApMRK-VirB6-3      KLDSDYIAPGSGRLYLAYWPYFGELGKKVKERKGSEAAVVAASGGSSQRGLTVSRAKNYSENSLLSALAQRRFFWKQDMSSGSGGFLSSNDAGNTYVPLS
ApNorV2-VirB6-3    KLDSDYIAPGSGRLYLAYWPYFGELGKKVKERKGREATVAAASGSSSQSGLTVSRAKNYSENSLLSALAQRRFFWKQDMSSGSGGFLSSNDAGNTYVPLS
ApVar1-VirB6-3     KLDSDYIAPGSGRLYLAYWPSLGELGKKVKERRENNA--VAASGSSSQRGLTVSRAKNYSENSLLSALAQRRFFWKQYMSFASGGFLPSNDAGNAYVSLS
ApNorV1-VirB6-3    KLDSDYIAPGSGRLYLAYWPSLGELGKKVKERKESKAAVVVASGGSSQRGSTVSSTKKYYEDSLLSALAQRRFFWKQDMSFASGGFLPSNDAGNAYVSLS
Consensus/80%      KLDSDYIAPGSGRLYLAYWP.bGELGKKVKERK.scAsVVAASGtSSQRGLTVSRAKNYSENSLLSALAQRRFFWKQsMS.tSGGFLsSNDAGNsYVsLS

ApHZ-VirB6-3       RDEGITTISYS-VASAATPVSAGKPASERRISVARTGEDIA-VQGFYSLNVHRTCYATSGQKLYMYIGDTPPTALPGKQQGAIPLDFEKINSSKS-DKEE
ApDog1-VirB6-3     RDEGITTISYS-VASAATPVSAGKPASERRISVARTGEDIA-VQGFYSLNVHRTCYATSGQKLYMYIGDTPPTALPGKQQGAIPLDFEKINSSKS-DKEE
ApDog2-VirB6-3     RDEGITTISYS-VASAATPVSAGKPASERRISVARTGEDIA-VQGFYSLNVHRTCYATSGQKLYMYIGDTPPTALPGKQQGAIPLDFEKINSSKS-DKEE
ApJM-VirB6-3       RDEGITTISYS-VASAATPVSAGKPASERRISVARTGEDIA-VQGFYSLNVHRTCYATSGQKLYMYIGDTPPTALPGKQQGAIPLDFEKINSSKS-DKEE
ApMRK-VirB6-3      RDEGITTISYS-VASAATPVSAGKPASERRISVARTGEDIA-VQGFYSLNVHRTCYATSGQKLYMYIGDTPPTALPGKQQGAIPLDFEKINSSKS-DKEA
ApNorV2-VirB6-3    RDEGITTISYS-IASAAMPVSAGNTASERRISVARTGEDIA-VQGFYSLNVHRTCYATSGQKLYMYIGDTPPTALPGKQLGAIPLDFEKINSSKG-DKEE
ApVar1-VirB6-3     HDEGATTISYSTVAAAAAPVSAGKTASQARMSVAHTSENTTKVQGFYALNVHRTCYATSGQKLYMYIGDTPPTALPGKQQGAIPLDFEKINSSKSDDKEE
ApNorV1-VirB6-3    HDEGATTISYSTVAAAAAPVSAGKTVSQSHISVARNSENTT-VQGFYSLSVRRTCYATSGQRLYMYIGDTPPTELPGKQTGAISLDFEEMNKS----KKE
Consensus/80%      +DEGhTTISYS.VAtAAsPVSAGKsASppRISVARTtEshs.VQGFYSLNVHRTCYATSGQKLYMYIGDTPPTALPGKQpGAIPLDFEKINSSKt.DKEE

ApHZ-VirB6-3       KEWSYKINSGAEKRQ-GYIYFGVDVDPGYEAKLKQANNSDNYYAVHLWVPKWTPIFSSFFNFLQGVLLHVLYGTDLPTMGQDTKAVEASKVIGRAMSPEY
ApDog1-VirB6-3     KEWSYKINSGAEKRQ-GYIYFGVDVDPGYEAKLKQANNSDNYYAVHLWVPKWTPIFSSFFNFLQGVLLHVLYGTDLPTMGQDTKAVEASKVIGRAMSPEY
ApDog2-VirB6-3     KEWSYKINSGAEKRQ-GYIYFGVDVDPGYEAKLKQANNSDNYYAVHLWVPKWTPIFSSFFNFLQGVLLHVLYGTDLPTMGQDTKAVEASKVIGRAMSPEY
ApJM-VirB6-3       KEWSYKINSGAEKRQ-GYIYFGVDVDPGYEAKLKQANNSDNYYAVHLWVPKWTPIFSSFFNFLQGVLLHVLYGTDLPTMGQDTKAVEASKVIGRAMSPEY
ApMRK-VirB6-3      KEWSYKINSGAEKRQ-GYIYFGVDVDPGYEAKLKQANNSDNYYAVHLWVPKWTPIFSSFFNFLQGVLLHVLYGTDLPTMGQDTKAVEASKVIGRAMSPEY
ApNorV2-VirB6-3    KEWSYKINSGAKKQQ-GYIYFGVDVDPGYEAKLKQANNSDNYYAVHLWVPKWTPIFSSFFNFLQGVLLHVLYGTDLPTMGQDTKAVEASKVIGRAMSPEY
ApVar1-VirB6-3     KEWSYKINSGAEKRQ-GYIYFGVDVDPGYEAKLKQANNSDNYYAVHLWVPKWTPIFSSFFNFLQGVLLHVLYGTDLPTMGQDTKAVEASKVIGRAMSPEY
ApNorV1-VirB6-3    KEWSYKINADKDHKQKGYIYFGVAVDPAYEANLKKASNTENYYSVHLWVPKWTPIFSSFFNLLQGMLLHVLYGTDLPTMGQDTRAVEAAKAIGKVMSPEY
Consensus/80%      KEWSYKINSGA-K+Q.GYIYFGVDVDPGYEAKLKQANNSDNYYAVHLWVPKWTPIFSSFFNFLQGVLLHVLYGTDLPTMGQDTKAVEASKVIGRAMSPEY
```

cont. Fig. S1

```
ApHZ-VirB6-3        IGIQGGGQQKKAGVVQQIYNNQVSTKPFWFAVRALLVLYLMFSVLGYIIGIIQVTKHDIFVRIAKIALIITLVSPGSWKFFTEHCFSIFILGIPDIISAF
ApDog1-VirB6-3      IGIQGGGQQKKAGVVQQIYNNQVSTKPFWFAVRALLVLYLMFSVLGYIIGIIQVTKHDIFVRIAKIALIITLVSPGSWKFFTEHCFSIFILGIPDIISAF
ApDog2-VirB6-3      IGIQGGGQQKKAGVVQQIYNNQVSTKPFWFAVRALLVLYLMFSVLGYIIGIIQVTKHDIFVRIAKIALIITLVSPGSWKFFTEHCFSIFILGIPDIISAF
ApJM-VirB6-3        IGIQGGGQQKKAGVVQQIYNNQVSTKPFWFAVRALLVLYLMFSVLGYIIGIIQVTKHDIFVRIAKIALIITLVSPGSWKFFTEHCFSIFILGIPDIISAF
ApMRK-VirB6-3       IGIQGGGQQKKAGVVQQIYNNQVSTKPFWFAVRALLVLYLMFSVLGYIIGIIQVTKHDIFVRIAKIALIITLVSPGSWKFFTEHCFSIFILGIPDIISAF
ApNorV2-VirB6-3     IGIQGGGQQKKAGVVQQIYNNQVSTKPFWFAVRALLVLYLMFSVLGYIIGIIQVTKHDIFVRIAKIALIITLVSPGSWKFFTEHCFSIFILGIPDIISAF
ApVar1-VirB6-3      IGIQGGEQQKKAGVVQQIYNNQVSTKPFWFAVRALLVLYLMFSVLGYIIGIIQVTKHDIFVRIAKIALIITLVSPGSWKFFTEHCFSIFILGIPDIISAF
ApNorV1-VirB6-3     IGVQGSG-KKKTGAVQQIYNNQVLTKPFWAAVRALLVLYLMFSVLGYIIGVIQVTKYDVFVRVAKIALIVTLVSPGSWQFFTEHCFSIFILGIPDIISAF
Consensus/80%       IGIQGGGQQKKAGVVQQIYNNQVSTKPFWFAVRALLVLYLMFSVLGYIIGIIQVTKHDIFVRIAKIALIITLVSPGSWKFFTEHCFSIFILGIPDIISAF

ApHZ-VirB6-3        NGYLGGDSSFAFLDSTLGIMLTSEFWLRMLSLFMAGPVGWLAFIGIIWALFSFFLAMMRAIILYLFIMVGLAFLLTLAPIFITFLLFQVTKGLFDGWLKM
ApDog1-VirB6-3      NGYLGGDSSFAFLDSTLGIMLTSEFWLRMLSLFMAGPVGWLAFIGIIWALFSFFLAMMRAIILYLFIMVGLAFLLTLAPIFITFLLFQVTKGLFDGWLKM
ApDog2-VirB6-3      NGYLGGDSSFAFLDSTLGIMLTSEFWLRMLSLFMAGPVGWLAFIGIIWALFSFFLAMMRAIILYLFIMVGLAFLLTLAPIFITFLLFQVTKGLFDGWLKM
ApJM-VirB6-3        NGYLGGDSSFAFLDSTLGIMLTSEFWLRMLSLFMAGPVGWLAFIGIIWALFSFFLAMMRAIILYLFIMVGLAFLLTLAPIFITFLLFQVTKGLFDGWLKM
ApMRK-VirB6-3       NGYLGGDSSFAFLDSTLGIMLTSEFWLRMLSLFMAGPVGWLAFIGVIWALFSFFLAMMRAIILYLFIMVGLAFLLTLAPIFITFLLFQVTKGLFDGWLKM
ApNorV2-VirB6-3     NGYLGGDSSFAFLDSTLGIMLTSEFWLRMLSLFMAGPVGWLAFIGIIWALFSFFLAMMRAIILYLFIMVGLAFLLTLAPIFITFLLFQVTKGLFDGWLKM
ApVar1-VirB6-3      NGYLGGDSSFAFLDSTLGIMLTSEFWLRMLSLFMAGPVGWLAFIGIIWALFSFFLAMMRAIILYLFIMVGLAFLLTLAPIFITFLLFQVTKGLFDGWLKM
ApNorV1-VirB6-3     NGYLGGDTSFAFLDSTLGIMLTSEFWLRMLSLFMAGPVGWLAFIGVIWALFSFFLAMMRAIILYLFVMVGLAFLLTLAPIFITFLLFQITKGLFDGWLKM
Consensus/80%       NGYLGGDSSFAFLDSTLGIMLTSEFWLRMLSLFMAGPVGWLAFIGlIWALFSFFLAMMRAIILYLFIMVGLAFLLTLAPIFITFLLFQVTKGLFDGWLKM

ApHZ-VirB6-3        LVNFMLQPIILFAALAFLNQVIITSLHAVTDFAACESCAVGFNISSKDSKAAPGQSDICIIPALLPMGYAFELPVSDRIREGLARGDIGFMGLPFSMAML
ApDog1-VirB6-3      LVNFMLQPIILFAALAFLNQVIITSLHAVTDFAACESCAVGFNISSKDSKAAPGQSDICIIPALLPMGYAFELPVSDRIREGLARGDIGFMGLPFSMAML
ApDog2-VirB6-3      LVNFMLQPIILFAALAFLNQVIITSLHAVTDFAACESCAVGFNISSKDSKAAPGQSDICIIPALLPMGYAFELPVSDRIREGLARGDIGFMGLPFSMAML
ApJM-VirB6-3        LVNFMLQPIILFAALAFLNQVIITSLHAVTDFAACESCAVGFNISSKDSKAAPGQSDICIIPALLPMGYAFELPVSDRIREGLARGDIGFMGLPFSMAML
ApMRK-VirB6-3       LVNFMLQPIILFAALAFLNQVIITSLHAVTDFAACESCAVGFNISSKDSKAAPGQSDICIIPALLPMGYAFELPVSDRIREGLARGDIGFMGLPFSMAML
ApNorV2-VirB6-3     LVNFMLQPIILFAALAFLNQVIITSLHAVTDFAACESCAVGFNISSKDSKAAPGQSDICIIPALLPMGYAFELPVSDRIREGLARGDIGFMGLPFSMAML
ApVar1-VirB6-3      LVNFMLQPVILFAALAFLNQVIITSLHAVTDFAACESCAVGFNISSKDSKAAPGQSDICIIPALLPMGYAFELPVSDRIREGLARGDIGFMGLPFSMAML
ApNorV1-VirB6-3     LVNFMLQPIILFAALAFLNQVIITSLHAVTDFAACEACAVGFNIPSKDSKAGPGQSDICIIPALLPTGFALDLPVSDRIREGLARGDIGFMGLPFSMAML
Consensus/80%       LVNFMLQPIILFAALAFLNQVIITSLHAVTDFAACESCAVGFNISSKDSKAAPGQSDICIIPALLPMGYAFELPVSDRIREGLARGDIGFMGLPFSMAML

ApHZ-VirB6-3        MVLILACKATREFGDIAEVMAHSISGSMSGMTAAAVGATQSMLSVVGLDDATQHLIRSAVAMDPVASDKVRFDAEDSVQPRHDGVK----DNSGADSPGK
ApDog1-VirB6-3      MVLILACKATREFGDIAEVMAHSISGSMSGMTAAAVGATQSMLSVVGLDDATQHLIRSAVAMDPVASDKVRFDAEDSVQPRHDGVK----DNSGADSPGK
ApDog2-VirB6-3      MVLILACKATREFGDIAEVMAHSISGSMSGMTAAAVGATQSMLSVVGLDDATQHLIRSAVAMDPVASDKVRFDAEDSVQPRHDGVK----DNSGADSPGK
ApJM-VirB6-3        MVLILACKATREFGDIAEVMAHSISGSMSGMTAAAVGATQSMLSVVGLDDATQHLIRSAVAMDPVASDKVRFDAEDSVQPRHDGVK----DNSGADSPGK
ApMRK-VirB6-3       MVLILACKATREFGDIAEVMAHSISGSVSGMTAAAVGATQSMLSVVGLDDATQHLIRSAVAMDPVASDKVRFDAEDSVQPRHDGVK----DNSGADSPGK
ApNorV2-VirB6-3     MVLILACKATREFGDIAEVMAHSISGSVSGMTAAAVGATQSMLSVVGLDDATQHLIRSAVAMDPVASDKVRFDAEDSVQPRHDGVK----DNSGADSPGK
ApVar1-VirB6-3      MVFILACKATREFGDIAEVMAHSISGSVSGMTAAAVGATQSMLSVVGLDDATQHLIRSAVAMDPVASDKVRFDAEDSVQPRHDGVK----DNSGADSPGK
ApNorV1-VirB6-3     MVLILACKATREFGDIAEVMAHSISGSVSGVTSAAVGATQSMLSVVGLDDATQHLIKSAIAMDPVGTDKVRFDAEDSVQPRHDGIKGPVDGSSGADSLGK
Consensus/80%       MVLILACKATREFGDIAEVMAHSISGShSGMTAAAVGATQSMLSVVGLDDATQHLIRSAVAMDPVASDKVRFDAEDSVQPRHDGVK....DNSGADSPGK
```

cont. Fig. S1

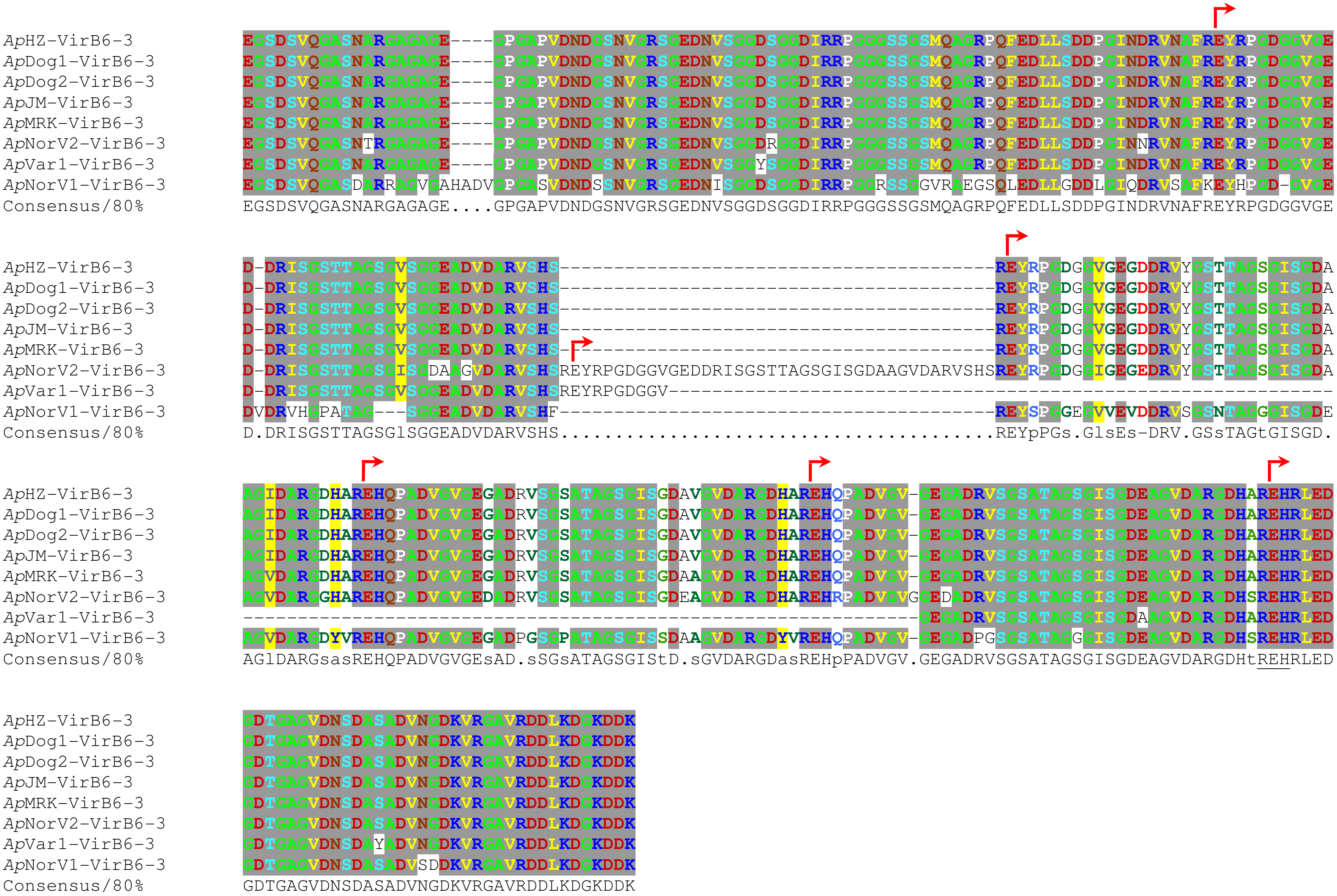
ApHZ-VirB6-3 EGSDSVQGASNARGAGAGE----GPGAPVDNDGSNVGRSGEDNVSGGDSGGDIRRPGGGSSGSMQAGRPQFEDLLSDDPGINDRVNAFREYRPGDGGVGE
ApDog1-VirB6-3 EGSDSVQGASNARGAGAGE----GPGAPVDNDGSNVGRSGEDNVSGGDSGGDIRRPGGGSSGSMQAGRPQFEDLLSDDPGINDRVNAFREYRPGDGGVGE
ApDog2-VirB6-3 EGSDSVQGASNARGAGAGE----GPGAPVDNDGSNVGRSGEDNVSGGDSGGDIRRPGGGSSGSMQAGRPQFEDLLSDDPGINDRVNAFREYRPGDGGVGE
ApJM-VirB6-3 EGSDSVQGASNARGAGAGE----GPGAPVDNDGSNVGRSGEDNVSGGDSGGDIRRPGGGSSGSMQAGRPQFEDLLSDDPGINDRVNAFREYRPGDGGVGE
ApMRK-VirB6-3 EGSDSVQGASNARGAGAGE----GPGAPVDNDGSNVGRSGEDNVSGGDSGGDIRRPGGGSSGSMQAGRPQFEDLLSDDPGINDRVNAFREYRPGDGGVGE
ApNorV2-VirB6-3 EGSDSVQGASNTRGAGAGE----GPGAPVDNDGSNVGRSGEDNVSGGDRGGDIRRPGGGSSGSMQAGRPQFEDLLSDDPGINNRVNAFREYRPGDGGVGE
ApVar1-VirB6-3 EGSDSVQGASNARGAGAGE----GPGAPVDNDGSNVGRSGEDNVSGGYSGGDIRRPGGGSSGSMQAGRPQFEDLLSDDPGINDRVNAFREYRPGDGGVGE
ApNorV1-VirB6-3 EGSDSVQGASDARRAGVGAHADVGPGASVDNDSSNVGRSGEDNISGGDSGGDIRRPGGRSSGGVRAEGSQLEDLLGDDLGIQDRVSAFKEYHPGD-GVGE
Consensus/80% EGSDSVQGASNARGAGAGE....GPGAPVDNDGSNVGRSGEDNVSGGDSGGDIRRPGGGSSGSMQAGRPQFEDLLSDDPGINDRVNAFREYRPGDGGVGE

ApHZ-VirB6-3 D-DRISGSTTAGSGVSGGEADVDARVSHS--REYRPGDGGVGEGDDRVYGSTTAGSGISGDA
ApDog1-VirB6-3 D-DRISGSTTAGSGVSGGEADVDARVSHS--REYRPGDGGVGEGDDRVYGSTTAGSGISGDA
ApDog2-VirB6-3 D-DRISGSTTAGSGVSGGEADVDARVSHS--REYRPGDGGVGEGDDRVYGSTTAGSGISGDA
ApJM-VirB6-3 D-DRISGSTTAGSGVSGGEADVDARVSHS--REYRPGDGGVGEGDDRVYGSTTAGSGISGDA
ApMRK-VirB6-3 D-DRISGSTTAGSGVSGGEADVDARVSHS--REYRPGDGGVGEGDDRVYGSTTAGSGISGDA
ApNorV2-VirB6-3 D-DRISGSTTAGSGISGDAAGVDARVSHSREYRPGDGGVGEDDRISGSTTAGSGISGDAAGVDARVSHSREYRPGDGGIGEGEDRVYGSTTAGSGISGDA
ApVar1-VirB6-3 D-DRISGSTTAGSGVSGGEADVDARVSHSREYRPGDGGV---
ApNorV1-VirB6-3 DVDRVHGPATAG---SGGEADVDARVSHF--REYSPGGEGVVEVDDRVSGSNTAGGGISGDE
Consensus/80% D.DRISGSTTAGSGlSGGEADVDARVSHS..REYpPGs.GlsEs-DRV.GSsTAGtGISGD.

ApHZ-VirB6-3 AGIDARGDHAREHQPADVGVGEGADRVSGSATAGSGISGDAVGVDARGDHAREHQPADVGV-GEGADRVSGSATAGSGISGDEAGVDARGDHAREHRLED
ApDog1-VirB6-3 AGIDARGDHAREHQPADVGVGEGADRVSGSATAGSGISGDAVGVDARGDHAREHQPADVGV-GEGADRVSGSATAGSGISGDEAGVDARGDHAREHRLED
ApDog2-VirB6-3 AGIDARGDHAREHQPADVGVGEGADRVSGSATAGSGISGDAVGVDARGDHAREHQPADVGV-GEGADRVSGSATAGSGISGDEAGVDARGDHAREHRLED
ApJM-VirB6-3 AGIDARGDHAREHQPADVGVGEGADRVSGSATAGSGISGDAVGVDARGDHAREHQPADVGV-GEGADRVSGSATAGSGISGDEAGVDARGDHAREHRLED
ApMRK-VirB6-3 AGVDARGDHAREHQPADVGVGEGADRVSGSATAGSGISGDAAGVDARGDHAREHQPADVGV-GEGADRVSGSATAGSGISGDEAGVDARGDHAREHRLED
ApNorV2-VirB6-3 AGVDARGGHAREHQPADVGVGEDADRVSGSATAGSGISGDEAGVDARGDHAREHRPADVGVGGEDADRVSGSATAGSGISGDEAGVDARGDHSREHRLED
ApVar1-VirB6-3 --GEGADRVSGSATAGSGISGDAAGVDARGDHAREHRLED
ApNorV1-VirB6-3 AGVDARGDYVREHQPADVGVGEGADPGSGPATAGSGISSDAAGVDARGDYVREHQPADVGV-GEGADPGSGSATAGGGISGDEAGVDARGDHSREHRLED
Consensus/80% AGlDARGsasREHQPADVGVGEsAD.sSGsATAGSGIStD.sGVDARGDasREHpPADVGV.GEGADRVSGSATAGSGISGDEAGVDARGDHtREHRLED

ApHZ-VirB6-3 GDTGAGVDNSDASADVNGDKVRGAVRDDLKDGKDDK
ApDog1-VirB6-3 GDTGAGVDNSDASADVNGDKVRGAVRDDLKDGKDDK
ApDog2-VirB6-3 GDTGAGVDNSDASADVNGDKVRGAVRDDLKDGKDDK
ApJM-VirB6-3 GDTGAGVDNSDASADVNGDKVRGAVRDDLKDGKDDK
ApMRK-VirB6-3 GDTGAGVDNSDASADVNGDKVRGAVRDDLKDGKDDK
ApNorV2-VirB6-3 GDTGAGVDNSDASADVNGDKVRGAVRDDLKDGKDDK
ApVar1-VirB6-3 GDTGAGVDNSDAYADVNGDKVRGAVRDDLKDGKDDK
ApNorV1-VirB6-3 GDTGAGVDNSDASADVSDDKVRGAVRDDLKDGKDDK
Consensus/80% GDTGAGVDNSDASADVNGDKVRGAVRDDLKDGKDDK
