# Supplementary material for: Structure of the type IV secretion system in different strains of Anaplasma phagocytophilum
Source: BMC Genomics. 2012 Nov 29;13:678. doi: 10.1186/1471-2164-13-678 (PMC3556328; doi:10.1186/1471-2164-13-678)
Supplement: Additional file 2 — Figure S2. Structure of the virB6-4 repeat regions R3 and R4 in four US A.phagocytophilum strains.A. Comparative maps of AB1393/AB1466 PCR products detailing the repeat unit content of R3 and R4 in the human, rodent and dog strains. ApJM and ApDog1 have identical virB6-4 genes and are, therefore, represented by one map. Moderate variability in the number and sequence of the R3 405 bp repeat units (light blue arrows) is apparent. The small bar at the end of R3 corresponds to the 3′-most partial repeat unit present in all strains. The colored arrows within R4 represent the five repeat types T1a (yellow), T1b (green), T2a, (red), T2b (dark blue) and T2c (grey). The repeat pattern in ApHZ shows no relationship to that of ApJM/ApDog1, which is also 2.87 kb larger, totaling 9.76 kb. This region was not fully characterized in ApDog2 as indicated by a broken line, but the repeat pattern of the 5′- and 3′-most repeats is clearly different from that of the other strains. The small bar downstream of the second repeat unit represents a partially characterized type 2 repeat unit. Lines above and below the ApHZ and ApJM/ApDog1 maps delineate segments of sequence identity within the respective R4 regions. Their sizes are specified. B. Alignment of the nucleic acid sequence of all virB6-4 R4 repeat unit types identified to date. Type 1 repeats are shown in black, type 2 in blue. Differences between sub-types are highlighted. A single BamHI site present in all type 2 repeats is underlined. With the exception of only a few nucleotides at each end, type 1 and type 2 repeat units do not share any sequences. C. Alignment of the amino acid sequences of the repeat units shown in B. The single nucleotide differences between sub-types do not lead to changes in amino acid sequence. [file 1471-2164-13-678-S2.pdf]

A.

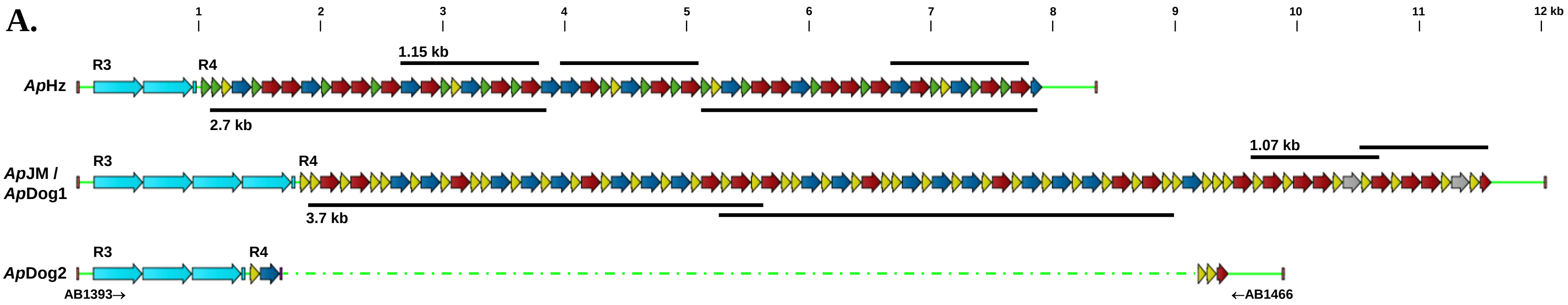

B.

```
T1a  CAGGAGAAGGAT---GGTAATACTGCGACGGAA---CT---TCCT-----AGAGAA--GT 44
T1b  CAGGAGAAGGAT---GGTAATACTGCGACGGAA---CT---TCCT-----AGAGAA--GT 44
T2a  CAGGATGGTGATAAGGGTGATTTAAGGCCTGAAAGACTGGATCCTGATATAGGGGATGGT 60
T2b  CAGGATGGTGATAAGGGTGATTTAAGGCCTGAAAGGCTGGATCCTGATATAGGGGATGGT 60
T2c  CAGGATGGTGATAAGGGTGATTTAAGGCCTGAAAGGCTGGATCCTGATATAGGGGATGGT 60
     *****    ***   *** **     * * ***   **   ****     ** * *  **

T1a  AGTAC------------------------------------------------------ 84
T1b  AGTAC------------------------------------------------------ 84
T2a  AGTGCTATAGAAGATGAGGTTGAGGTTAGATCTTCTAGGAGTAGTGAATCAACTGATAG 119
T2b  AGTGCTATAGAAGATGAGGTTGAGGTTAGATCTTCTAGGAGTAGTGAATCAACTGATAG 119
T2c  AGTGCTATAGAAGATGAGGTTGAGGTTAGATCTTCTAGGAGTAGTGAATCAACTGATAG 119
     *** *

T1a  --------CTGAAGCTACCGAGTATGGTACTAAACCTGATGAT 84
T1b  --------CTGAAGCTACTGAGTATGGTACTAAACCTGATGAT 84
T2a  CGTGCCATCTGAAGTAACTGAACGTGATGCTCAACGTGATGAT 162
T2b  CGTGCCATCTGAAGTAACTGAACGTGATGCTCAACGTGATGAT 162
T2c  TGTGCCATCTGAAGTAACTGAA------------CGTGATGAT 150
             ******  ** **   ** * ** *** *******
```

C.

```
T1a/T1b  QEKD--------------------------GNTATELPREVVPEATEYGTKPDD 28

T2a/T2b  QDGDKGDLRPERLDPDIGDGSAIEDEVEVRSSRSSESTDSVPSEVTERDAQRDD 54
T2c      QDGDKGDLRPERLDPDIGDGSAIEDEVEVRSSRSSESTDSVPSEVTE----RDD 50
         *: *                          .. ::* . .* .*.**     **
```
